# Polyketide synthesis genes associated with toxin production in two species of *Gambierdiscus* (Dinophyceae)

**DOI:** 10.1186/s12864-015-1625-y

**Published:** 2015-05-28

**Authors:** Gurjeet S. Kohli, Uwe John, Rosa I. Figueroa, Lesley L. Rhodes, D. Tim Harwood, Marco Groth, Christopher J. S. Bolch, Shauna A. Murray

**Affiliations:** Plant Functional Biology and Climate Change Cluster, University of Technology Sydney, Broadway, New South Wales 2007 Australia; School of Biotechnology and Biomolecular Sciences, University of New South Wales, Kensington, New South Wales 2052 Australia; Alfred Wegener Institute for Polar and Marine Research, Bremerhaven, 27515 Germany; Aquatic Ecology, Department of Biology, Lund University, Lund, 223 62 Sweden; Cawthron Institute, Nelson, 7010 New Zealand; Leibniz Institute for Age Research, Fritz Lipmann Institute, Jena, D-07745, Germany; Institute for Marine and Antarctic Studies, University of Tasmania, Launceston, 7248 Tasmania Australia; Sydney Institute of Marine Sciences, Mosman, New South Wales 2088 Australia

## Abstract

**Background:**

Marine microbial protists, in particular, dinoflagellates, produce polyketide toxins with ecosystem-wide and human health impacts. Species of *Gambierdiscus* produce the polyether ladder compounds ciguatoxins and maitotoxins, which can lead to ciguatera fish poisoning, a serious human illness associated with reef fish consumption. Genes associated with the biosynthesis of polyether ladder compounds are yet to be elucidated, however, stable isotope feeding studies of such compounds consistently support their polyketide origin indicating that polyketide synthases are involved in their biosynthesis.

**Results:**

Here, we report the toxicity, genome size, gene content and transcriptome of *Gambierdiscus australes* and *G. belizeanus. G. australes* produced maitotoxin-1 and maitotoxin-3, while *G. belizeanus* produced maitotoxin-3, for which cell extracts were toxic to mice by IP injection (LD_50_ = 3.8 mg kg^-1^). The gene catalogues comprised 83,353 and 84,870 unique contigs, with genome sizes of 32.5 ± 3.7 Gbp and 35 ± 0.88 Gbp, respectively, and are amongst the most comprehensive yet reported from a dinoflagellate. We found three hundred and six genes involved in polyketide biosynthesis, including one hundred and ninty-two ketoacyl synthase transcripts, which formed five unique phylogenetic clusters.

**Conclusions:**

Two clusters were unique to these maitotoxin-producing dinoflagellate species, suggesting that they may be associated with maitotoxin biosynthesis. This work represents a significant step forward in our understanding of the genetic basis of polyketide production in dinoflagellates, in particular, species responsible for ciguatera fish poisoning.

**Electronic supplementary material:**

The online version of this article (doi:10.1186/s12864-015-1625-y) contains supplementary material, which is available to authorized users.

## Background

**Fig. 1:**
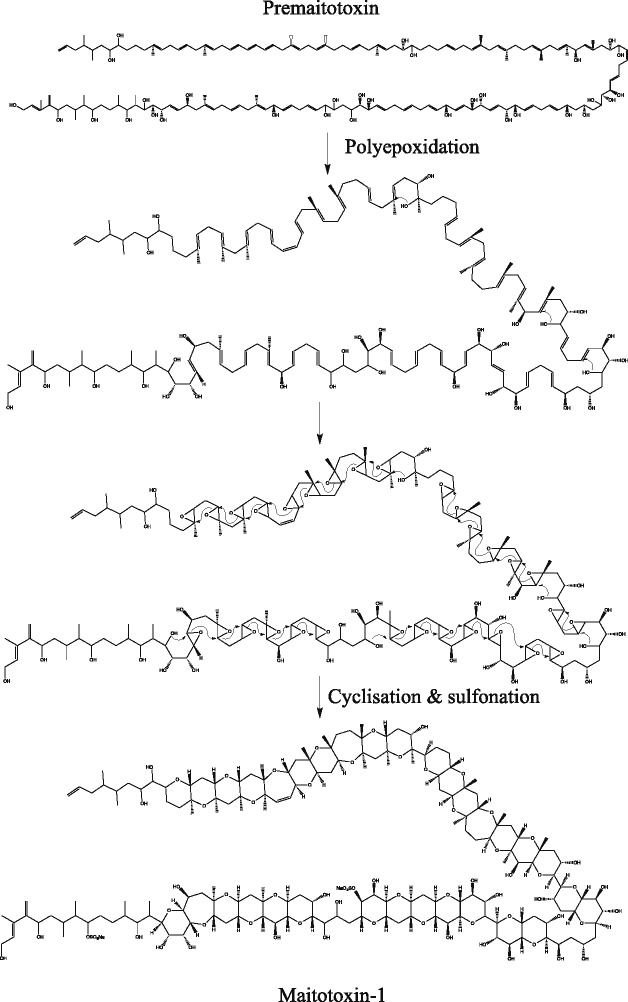
Proposed mechanism for maitotoxin-1 production. Polyene (premaitotoxin) produced by polyketide biosynthesis undergoes epoxidation, epoxide cyclisation and sulfonation to form Maitotoxin-1

*Gambierdiscus* species (Dinophyceae) can produce maitotoxins (MTXs), ladder-like polycyclic ether compounds [1, 2], a structure principally reported from dinoflagellates. MTX-1 is the largest and the most toxic natural non-biopolymer known [1, 2], and is similar to other polyether compounds such as okadaic acid (OA) and brevetoxins (BTXs) produced by *Prorocentrum* spp and *Karenia brevis*, respectively (reviewed [3, 4]). A plethora of stable isotope feeding studies conducted on dinoflagellates producing BTXs, OA and dinophysistoxins (DTXs) provide substantial evidence to support the polyketide origin of these polyether ladder compounds [3, 5–9]. Despite this, gene clusters associated with the biosynthesis of polyether ladders have not been elucidated, and little is known about the genes involved in this process. Gene clusters responsible for the biosynthesis of the non-ladder polyether compounds, monensin and nanchangmycin, have been elucidated in bacteria [10, 11]. The putative alkene precursor of these compounds are synthesised via type I polyketide synthases (PKS). It is proposed that the alkene undergoes epoxidation and polyepoxide cyclisation to form ether linkages [12]. In monensin biosynthesis, these steps could be performed by putative epoxidases and epoxide hydrolases, which are also found in the gene cluster responsible for monensin biosynthesis in addition to a full type I PKS assembly [10, 12, 13]. In the case of monensin, the deletion of either of these genes ceases the production of the polyether, supporting this hypothesis [12]. In the case of BTXs, it is also proposed that the carbon backbone is mostly *trans*-polyene, and it undergoes epoxidation and polyepoxide cyclisation to form BTX [7, 14]. Very little evidence has been determined to support this hypothesis for BTXs, however, there is evidence suggesting the presence of beta epoxidation intermediate, shown by ^18^O incorporation from molecular oxygen into C, D and E rings of OA [9] and yessotoxins [15]. Similar to the proposed biosynthetic pathway for BTXs [7, 14], we propose a possible biosynthetic pathway for MTX-1 synthesis (Fig. 1), in which the carbon backbone is synthesised via polyketide biosynthesis followed by epoxidation, polyepoxide cyclisation and sulphonation carried out by PKSs, epoxidases, epoxide hydrolases and sulfotransferases.

There are three major functional groups of PKSs. Type I PKS are large multifunctional proteins, comprising several essential domains: acyltransferase domain (AT), β-ketosynthase domain (KS) and acyl carrier protein (ACP); they can also include β-ketoacyl reductase (KR), enoyl reductase (ER), methyl transferases (MT), thioesterases (TE) and dehydrogenase (DH) domains [16]. In iterative type I polyketide synthesis the same set of catalytic domains are present in one protein and used in a cyclic fashion for chain elongation, analogous to fatty acid synthesis (FAS) [17, 18]. In contrast, modular type I PKS catalytic domains are organised in modules comprising all required domains for each step and each module is only used once during polyketide assembly [16]. Type II PKS consist of mono-functional proteins with each catalytic domain on a separate peptide that form complexes for polyketide assembly [19].

Dinoflagellates possess some of the largest genomes known from eukaryotes, from 1.85 to 112 Gbp [20]. Their gene content has been estimated to be much smaller than would be anticipated based on their genome size, at 38,188 – 87,688 protein coding genes [21]. The copy number of individual genes can vary significantly, between 30 copies (protein kinase gene in *L. polyedrum*) [22] to 5000 copies (peridinin-chlorophyll a-binding protein gene studied in *L. polyedrum*) [23], and up to 100,000 copies of common genes such as rRNA [24]. Such huge genome sizes and high gene copy numbers have made entire genome sequencing for these organisms unfeasible. Recent advancement of high throughput sequencing technologies has now made it feasible to study the gene content of these organisms at the genomic and transcriptomic level. Recently, a partial draft assembly of *Symbiodinium minutum* genome, which has amongst the smallest genomes of a dinoflagellate, has been achieved [25]. In dinoflagellates, the lack of axenic cultures, as well as the difficulty in constructing genetic mutations and screening mutants, has meant that confirming the roles of genes in biosynthetic pathways is currently not possible. Despite the challenges, type I modular PKS genes have been identified in *Karenia brevis* [26, 27], *Heterocapsa circularisquama* [28], *Heterocapsa triquetra* [29], *Alexandrium ostenfeldii* [29], *Azadinium* sp. [30] and several *Amphidinium* species [31, 32] via transcriptomics. In dinoflagellates mRNA undergoes *trans*-splicing with an addition of a 22-nt conserved spliced leader (SL) to the 5’ end of the sequence [33]. Sequencing of full-length mature mRNA transcripts containing the SL sequence and phylogenetic analysis is necessary to distinguish these sequences from bacterial PKS genes, originating from non-axenic cultures. Interestingly, in most of the previous studies, full-length transcripts only encoded one catalytic domain, but were homologous to type I PKSs, suggesting a novel mono-functional type I PKS in dinoflagellates [29]. However, if polyether ladders are produced by modular type I PKS enzymes, based on the structure of these compounds, the PKS sequences discovered so far likely represent only a fraction of the PKS genes present.

Here we present comprehensive transcriptomic libraries of two species of gonyaulacaleaen and MTX producing dinoflagellates, *Gambierdiscus australes* and *G. belizeanus*. A large number of genes putatively involved in the biosynthesis of polyether ladder compounds were found. In addition, genes involved in other regulatory pathways were also mapped. Genome sizes and the number of genes were estimated using flow cytometry and statistical analysis. The toxin profiles of the species were generated via liquid chromatography- mass spectrometry (LC-MS) against toxin standards, and the toxicity was determined using mouse bioassay.

## Results and discussion

### Genome size analysis

**Fig. 2:**
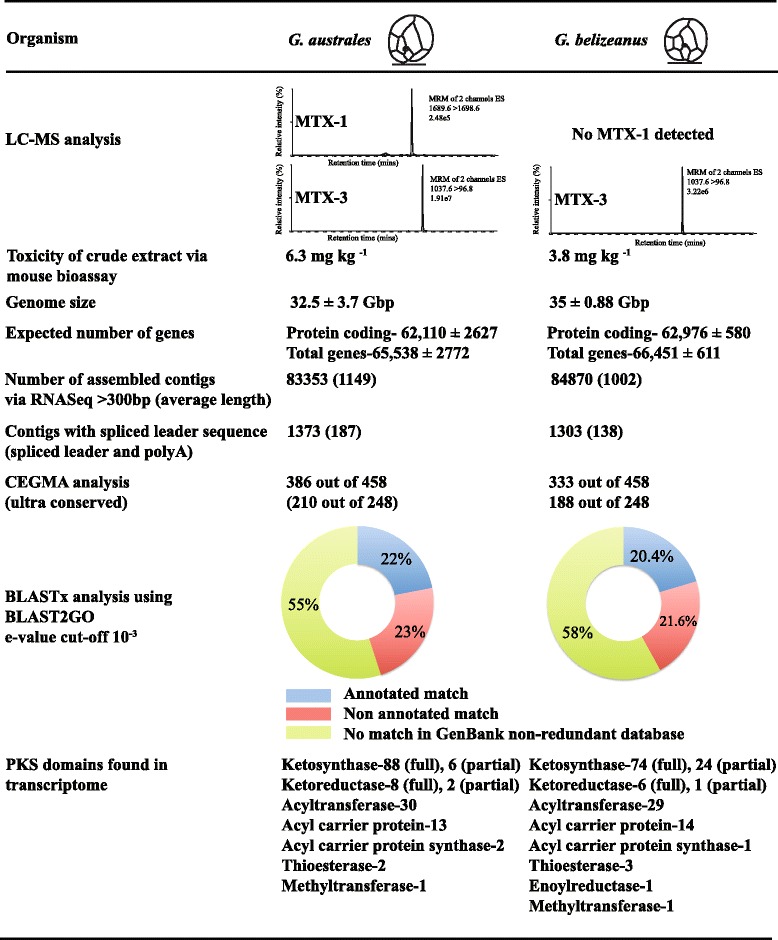
Genome sizes obtained via flow cytometry, chemical analyses via LC-MS, mouse bioassays and bioinformatic analysis of gene catalogues of *G. australes* and *G. belizeanus*

We determined a DNA content of 33.2 ± 3.8 pg cell^-1^ for *Gambierdiscus australes* and 35.8 ± 0.9 pg cell^-1^ for *G. belizeanus* via flow cytometry (Supplemental data), which equates to a genome size of 32.5 ± 3.7 Gbp and 35 ± 0.88 Gbp, respectively (Fig. 2). While very large compared to other eukaryotes, both genomes were comparatively smaller than expected, given the large cell sizes of *Gambierdiscus* species based on a comparison of 23 dinoflagellate species (Additional file 1: Figure S1 and Table S1). Genome sequencing has been used to elucidate PKS gene clusters from many organisms, however, instead of sequencing such large genomes, comparative transcriptomic studies may be an efficient method for finding novel dinoflagellate genes [34, 35].

### Toxin analysis

Cell extracts of *G. australes* CAWD149 contained MTX-1 and putative MTX-3, whereas *G. belizeanus* CCMP401 extract contained only putative MTX-3 (Fig. 2). No ciguatoxins were detected in extracts of either species. While the toxicity of MTX-1 to mice by IP injection is well documented [2], the toxicity of MTX-3 had not been previously determined. The cell extract of *G. belizeanus* had an LD_50_ of 3.8 mg kg^-1^ in mouse bioassays using IP injection, causing abdominal breathing, decreased respiration rates and ultimately death through respiratory paralysis. The LD_50_ of this extract is higher than that for pure MTX-1 (0.05 μg kg^-1^) [2], but lower than the LD_50_ of other toxins such as saxitoxin (8–10 μg kg^-1^) [36, 37]. The structure of MTX-3 is not yet fully elucidated, however, it probably has a polyether ladder structure similar to MTX-1 [2].

### Transcriptomic analysis

We generated comprehensive transcriptomic libraries of two species of MTX-producing and non-ciguatoxins (CTX) producing dinoflagellates, *Gambierdiscus australes* and *G. belizeanus,* with an aim to investigate the evolution of PKS enzyme complexes (for details, see experimental procedure in supplemental data). Here, we report gene catalogues of 83,353 (*G. australes*) and 84,870 (*G. belizeanus*) unique contigs, that are amongst the most comprehensive yet reported from dinoflagellates (Fig. 2). Based on the genome size, gene number was estimated as 62,110 ± 2627 protein-coding and 65,538 ± 2772 total genes per genome in *G. australes*, and 62,976 ± 580 protein-coding and 66,451 ± 611 total genes per genome in *G. belizeanus* using the empirical regression equation of Hou & Lin [21]. Sequences encoding all the essential enzymes involved in glycolysis, tricarboxalic acid cycle, C-3 carbon cycle, pentose phosphate pathway and oxidative phosphorylation were found and could be fully annotated among the 18,399 and 17,290 fully annotated sequences in *G. australes* and *G. belizeanus* transcriptomes respectively (Additional file 1: Table S2).

The presence of 84.27 % (*G. australes*) and 72.7 % (*G. belizeanus*) of 458 highly conserved proteins included in the core eukaryotic genes mapping approach (CEGMA) software, served as an additional test of the comprehensiveness of the catalogues (Fig. 2, [38]). The other published protist gene catalogues investigated via CEGMA analysis to date, *Toxoplasma gondii*, had 67.63 % [38], *Karenia brevis* had 84 % (SP1), 82 % (SP3) and 81 % (Wilson) [27] and *Karlodinium micrum* had 74 % [39] of the 458 highly conserved protein datasets. However, some protein orthologues may not have been recognised using CEGMA analysis due to the high degree of sequence divergence of protists from other eukaryotes.

A full suite of histone encoding genes (H2A, H2B, H3, H4) was also found in both the gene catalogues (Additional file 1: Table S3) as previously reported in various *Symbiodinium* species [25, 40] and *Lingulodinium polyedrum* [41]. A phylogeny of the H2A histone proteins revealed the presence of H2A.X variants of the histone proteins. Dinoflagellate H2A.X sequences form a distinct well-supported clade and were clearly distinguished from other major groups of H2A.X and H2A.Z variants (Additional file 1: Figure S2).

In dinoflagellates, the presence of SL sequence provides a means to distinguish full-length mature dinoflagellate transcripts from transcripts derived from bacteria associated with non-axenic cultures. Our gene catalogue consisted of one of the largest collection of full-length transcripts (SL at 5’ end and polyA tail at 3’ end) reported for any dinoflagellate transcriptome library (Fig. 1, Additional file 1: Table S4). The fact that only 63 % and 54 % of full-length transcripts, respectively, could be annotated is intriguing, suggesting that many transcripts might be involved in novel processes. This was also the case for total transcripts in the gene catalogues, with more than 50 % having no BLASTx match, similar to recently published catalogues from *L. polyedrum* (total of 74,655 contigs, 25 % annotated matches, 45 % non-annotated matches and 30 % without similarity to any known sequences in GenBank [42]) and *K.brevis* (total of 43–45 % of transcripts belonging to three strains had a positive BLASTp match to the nr database [27]).

**Fig. 3:**
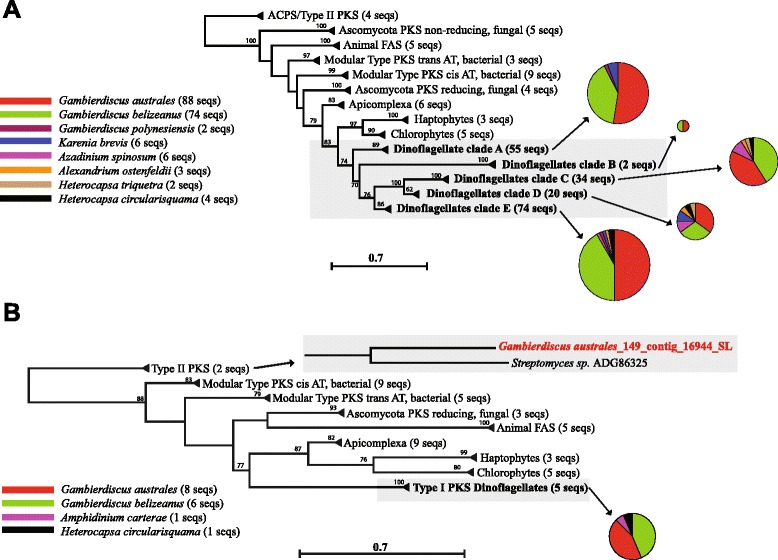
Phylogenetic analysis of polyketide synthases (PKS) genes. **a** Phylogenetic analysis of type I and type II ketoacyl synthase (KS) domains from prokaryotic and eukaryotic PKS and fatty acid synthases (FAS). Two hundred and twenty nine KS domains representing 38 taxa were analysed via a maximum likelihood approach in PhyML using the Le Gascuel substitution model and 100 bootstrap replicates. **b** Phylogenetic analysis of type I and type II ketoreductase (KR) domains. The sequences include prokaryotic and eukaryotic PKSs and FASs. Fifty-six KR domains representing 25 taxa were analysed via maximum likelihood approach using the Le Gascuel substitution model and 100 bootstrap replicates

Our *Gambierdiscus* gene catalogues contained a large number and diversity of genes putatively involved in polyketide biosynthesis, including a total of 162 unique transcripts (88 in *G. australes* and 74 in *G. belizeanus*) encoding complete KS domains (Fig. 2). The presence of dinoflagellate-specific SL in five KS transcripts, their similarity (BLASTx) to KS domains from other dinoflagellates (Additional file 1: Tables S5–S7) and the monophyletic clustering of all dinoflagellate KS transcripts within a protistan KS domain cluster in the phylogenies, provides consistent and substantial evidence of the dinoflagellate origin of these transcripts (Fig. 3a). The 185 dinoflagellate KS transcripts included in the phylogenetic analysis grouped with type I PKS with strong support and could be resolved in 5 well-supported clades within the dinoflagellate clade (Fig. 3a). As KS domains are used by PKSs and fatty acid synthases (FAS), the transcripts in these clades might be involved in either or both of these processes. The clades also consisted of KS transcripts found only in BTX-producing *Karenia brevis* [26, 27], including four KS transcripts found in both BTX-producing and non-producing *K. brevis* [26, 27], three KS transcripts from spirolide producing *Alexandrium ostenfeldii* [29], five KS transcripts from azaspiracid producing *Azadinium spinosum* [30], two KS transcripts from CTX producing *G. polynesiensis* [43], two KS transcripts from toxic *Heterocapsa triquetra* [29]and three KS transcripts from non-toxic *H. circularisquama* [28].

We suggest that clades C, D and E, which contain KS sequences from almost all dinoflagellates included in the analyses, are more likely to be involved in a common synthesis pathway, such as FAS. However, Clade A contained only KS sequences from polyether ladder-producing dinoflagellates while Clade B contained only KS sequences from *G. belizeanus* and *G. australes* (Fig. 3a), suggesting these sequences may be involved in the synthesis of MTXs or other unique PKS products from this genus. Most previous studies of KS diversity in dinoflagellate transcriptomes had much lower sequence depth and coverage, therefore similar sequences may be present in other species, but have not been detected. This includes the recently published *G. polynesiensis* transcriptome [43] in which 33 transcripts encoding KS domains were detected, however, only two transcripts had full-length KS domains encoded. In-depth transcriptomic analysis of cultures of other dinoflagellates with comprehensive toxin profiles are required to be more certain of the role of the sequences in these three clades.

Both *Gambierdiscus* transcriptomes examined here also contained numerous other putative polyketide domains (Fig. 1, Additional file 1: Tables S8–S9), including a transcript resembling a type II KR domain (Fig. 3b, a SL at the start of the transcript confirms its dinoflagellate origin). KR domains have been found previously in *K. brevis* [26, 27], *A. carterae* [31] and *A. spinosum* [30]. This is the first study to show that the majority form a strongly supported cluster with type I PKS (Fig. 3b).

As demonstrated previously [26, 29, 43], a surprising feature of the dinoflagellate transcripts encoding KS and KR domains is that they contain only one domain per transcript. Previously this feature has been characteristic of type II PKSs, yet our phylogenetic analyses show that they formed strongly supported clades with other type I PKS sequences (Fig. 3), except for the single type II related KR domain described above (Fig. 3b). Other protists such as species of apicomplexans and haptophytes display classic type I PKS modules, containing several domains, encoded on a single transcript [44, 45] and it appears that the monofunctional expression of type I PKS transcripts is unique to dinoflagellates [29, 30].

Some PKS AT domain transcripts also encoded ankyrin proteins (Additional file 1: Table S9), which are known to mediate the attachment of integral membrane proteins in mammals [46]. ATs with ankyrin proteins are generally involved in many other pathways [47] and often are not embedded into PK megasynthases, but instead operating as individual *trans* proteins [48]. Their variable genomic clustering therefore makes it difficult to identify, which ATs may be involved in polyketide synthesis [49].

If MTX-1 is produced by type I modular PKS it would likely contain 69 modules, given the monofunctional role of these enzymes. If each KS domain in each module was encoded by a separate transcript then this accounts for the large number of transcripts recovered from these two *Gambierdiscus* species. Early theoretical pathways for polyether ladder synthesis predict epoxidation and cyclisation of polyether ladders during polyketide synthesis [50], however, the pathway proposed here involves modification of the carbon backbone after polyketide synthesis (Fig. 1) as in the case of monensin biosynthesis, which is a non-ladder polyether compound [10, 12]. We also detected transcripts encoding for enzymes epoxidases, epoxy hydrolases and sulfotransferases that could perform expoxidation, cyclisation and sulfonation of polyether compounds, respectively (Additional file 1: Table S10), supporting the proposed biosynthesis.

## Conclusion

In dinoflagellates, the difficulty of generating and subsequent poor survival of axenic cultures, combined with the difficulty of genetic transformation and screening, means that confirming the role of genes in toxin biosynthesis pathways is currently very difficult. Linking genes to polyketide production in dinoflagellates requires comparative transcriptomic studies of species with contrasting polyketide production profiles. The results presented here are a major contribution towards eventually recognising the genes that encode a critical step in each type of polyketide biosynthesis.

## Methods

### *Gambierdiscus* cell culture

*Gambierdiscus australes* (CAWD149, originally isolated from Cook Islands, Pacific Ocean, kindly provided by Lesley Rhodes, Cawthron Institutes culture collection of Micro Algae) and *Gambierdiscus belizeanus* (CCMP401, originally isolated from Barthelemy Island, Caribbean Sea, North Atlantic Ocean, purchased from National Centre for Marine Algae and Microbiota) strains were cultured at 25 °C under cool white fluorescent light at a light intensity of 60 μmol m^-2^ s^-1^ and a 12:12 light:dark cycle. *G. australes* was grown in f/2 medium [51] *G. belizeanus* was grown in K medium [52].

### DNA and RNA extraction

For DNA and RNA extraction of *G. australes*, cells were harvested by separation over 3.0 μM filters (Merck Millipore, Darmstadt, Germany) and washed with Phosphate buffered saline (Sigma, St. Louis, MO) three times to minimise bacterial contamination.

For, DNA extraction the cell pellet was extracted via FastDNA® Spin kit for Soil (MP Biomedicals, Solon, OH). The manufacturer’s protocol was followed, and samples were stored at -20 °C until further analysis.

For, RNA extraction the cell pellet was first extracted via TriReagent® (Life Technologies, Carlsbad, CA) using the manufacturer’s protocol. The obtained RNA was purified using the RNeasy Plant mini kit (Qiagen, Limberg, Netherlands) according to the manufactures protocol. Any residual DNA was removed via the TURBO DNA-free™ Kit (Life Technologies) and RNA was stored at -80 °C until further analysis. The RNA purity, quantity and integrity were assessed using Nanodrop ND-1000 (Thermo Scientific, Woltham, MA) and 2100 Bioanalyser (Agilent Technologies, Santa Clara, CA).

### Toxin analysis via LC-MS and mouse bioassay

*G. australes* and *G. belizeanus* cell pellets were extracted using a previously standardised method for CTX [53] and MTX analysis [54]. The LC-MS analysis was performed at the Cawthron Institute, Nelson, New Zealand, with multiple-reaction monitoring for CTX-3b, CTX-3C, CTX-4A, CTX-4B, MTX-1 and MTX-3.

Mouse bioassays were conducted at Agri Research, Hamilton, New Zealand. To test the toxicity of MTX-3, a cell pellet of *G. belizeanus* containing 4.776 × 10^6^ cells extracted with methanol. The dried extract was dissolved in 1 % Tween 60 in saline and female Swiss albino mice (body weight 18–22 g) were injected intra-peritoneally with this solution at various dose levels. The LD_50_ values were determined by the up-and-down method [55].

### Genome size estimation via flow cytometry

Synchronisation and sample collection was achieved by inoculating *G. australes* and *G. belizeanus* cell cultures at an initial concentration of 1000 cells ml^-1^. The cells were grown for eight days and then synchronised via 48:48:48 h dark:light:dark cycle and then harvested via centrifugation at 1000 *g* for 5 min. The cell pellet was resuspended in 4 mL of methanol and stored until further analysis. For, flow cytometry, the collected cells were washed twice in PBS and the pellet was resuspended in a staining solution (PBS, 100 μg propidium iodide mL^-1^ and 2 μg RNaseA. mL^-1^) for at least 4 h before analysis. A Beckman FC500 bench flow cytometer (Beckman Coulter, Brea, CA) with a laser emitting at 488 nm was used. Three replicate samples for each species were run at low speed and data were acquired in linear and log modes until at least 1000 events had been recorded. As DNA standard, 10 μl of a triploid trout solution (7.8 pg/nucleus, Biosure, Grass valley, CA) was added to each sample. Fluorescence emission of propidium iodide was detected at 620 nm. FlowJo 7.6 (Tree Star Inc., Ashland, OR) was used to compute peak numbers, coefficients of variation (CVs), and peak ratios for the DNA fluorescence distributions in a population. CV values were typically less than 20 %. The genome size was calculated based on the conversion factor 1 pg = 978 Mbp [56].

To make valid predictions of gene numbers in the genome*,* the empirical regression equation y’ = ln(-46.200 + 22.217x’) and y’ = ln(247.28 + 22.74x’) provided by Hou & Lin [21] were used to calculate the predicted protein coding genes and total number of genes in a genome, respectively. In the equation y’ = Log_10_ gene number and x’ = Log_10_ genome size in kbp.

### Transcriptome analysis

RNA extracted from *G. australes* CAWD149 and *G. belizeanus* CCMP401 was sequenced using a HiSeq2000 (Illumina, San Diego, CA) generating 100 bp paired-end reads. The libraries were prepared using the TruSeq™ RNA Sample Prep Kit v2 following the manufacturer’s description (Illumina, order no. RS-122–200x) which involves selective polyA^+^ RNA enrichment using magnetic beads followed by fragmentation of enriched RNA fraction (for only 4 min to obtain also fragments with sizes > 300 bp), adapter ligation and amplification. For sequencing both libraries were multiplexed in one lane. A total of 79,265,976 and 61,587,248 read pairs were extracted in FastQ format using CASAVA v1.8.2 (Illumina) for *G. australes* and *G. belizaeanus*, respectively. Raw reads were quality filtered and assembled into contigs using the CLC Genomics Workbench (CLC bio, Cambridge, MA) and the software’s default settings. Any contigs with a length of less than 300 bp (based on the length of the insert size) were not analysed further. BLASTx analysis, mapping, annotation and Kyoto Encyclopedia of Gene and Genomes (KEGG) analysis for both the gene catalogues was performed using BLAST2GO [57]. BLASTx was performed against the nr database of GenBank and an E-value cut off of 10^-3^ was used. For mapping and annotations, the default values of BLAST2GO were used. To analyse the comprehensiveness of the gene catalogues the Core Eukaryotic Genes Mapping approach (CEGMA) tool was used [38]. Identification of potential genes involved in polyketide biosynthesis was achieved by text searching the annotations (ketosynthase, PKS, polyketide synthase, ketoreductases). For the identification of KS and KR domains, these sequences were further analysed by PKS-NRPS analysis software [58] and HMMER [59] (using an in-house developed HMM databases). Functional prediction of sequences was also aided by running Pfam [60] searches. To calculate the amount of sequences of bacterial origin, any sequences with a top BLASTx hit to prokaryotic organisms were counted.

For phylogenetic analysis, all steps were performed in Geneious® software [61]. Sequences from different datasets were aligned using MAFFT v6.814b [62]. Alignments were trimmed manually to ensure they spanned the same KS/KR/Histone2a coding region. After aligning the sequences, the best substitution model was determined using ModelTest [39] and a maximum likelihood phylogenetic analysis was carried out using the program PhyML [63] with 100 bootstraps.

## Additional file

Additional file 1: Figure S1. Correlation between cell dimensions (length of dorso-ventral axis of a cell) and quantity of nuclear DNA in 27 different species of dinoflagellates (23 genera). **Figure S2.** Phylogenetic analysis of H2A histone proteins. **Table S1.** Data used to estimate the correlation between cell dimensions and quantity of nuclear DNA in dinoflagellates (23 genera). **Table S2.** Description of different sequences from *G. australes* and *G. belizeanus* encoding essential enzymes for Glycolysis, TCA cycle, Oxidative phosphorylation, Carbon fixation (c3) cycle and pentose phosphate pathway. **Table S3.** A list of all the sequencing encoding the histone proteins found in the gene catalogue of *G. australes* and *G. belizeanus*. **Table S4.** A list of sequences from *G. australes* and *G. belizeanus* gene catalogue containing spliced leader at the 5’ end and polyA tail at the 3’ end. **Table S5.** Sequence properties of the transcripts encoding full ketoacyl synthase domain identified in *G. australes* CAWD149. **Table S6.** Sequence properties of the transcripts encoding full ketoacyl synthase domain identified in *G. belizeanus.* **Table S7.** Sequence properties of the transcripts encoding partial ketoacyl synthase domain identified in *G. australes* and *G. belizeanus*. **Table S8.** Sequence properties of the transcripts encoding full and partial ketoyl reductase domains identified in *G. australes* and *G. belizeanus*. **Table S9.** Sequence properties of the transcripts encoding full acyl carrier protein synthase, enoylreductase, acyltransferase, acyltransferase with ankyrin adaptor proteins, acyl carrier protein domains identified in *G. australes* and *G. belizeanus*. **Table 10.** Sequence properties of the transcripts encoding epoxidases, epoxide hydrolases and full and partial sulfotransferases enzymes identified in *G. australes* and *G. belizeanus*. **Table S11.** Sequence properties of the transcripts encoding full acyl carrier protein synthase, Enoyl reductase, acyl transferase, acyl carrier protein identified in *G. australes* and *G. belizeanus*. **Table S12.** Sequence properties of the transcripts encoding epoxide hydrolases and sulfotransferases enzymes identified in G. australes and *G. belizeanus*. **Table S13.** Sequence properties of the transcripts encoding multiple Type I PKS domains identified in *G. australes* and *G. belizeanus*.

## Abbreviations

(OA): Okadaic acid
(BTXs): Brevetoxins
(MTXs): Maitotoxins
(DTXs): Dinophysistoxins
(PKS): Polyketide synthases
(AT): Acyltransferase domain
(KS): β-ketosynthase domain
(ACP): Acyl carrier protein
(KR): β-ketoacyl reductase
(ER): Enoyl reductase
(MT): Methyl transferases
(TE): Thioesterases
(DH): Dehydrogenase
(FAS): Fatty acid synthesis
(SL): Spliced leader
(LC-MS): Liquid chromatography- mass spectrometry
(CEGMA): Core eukaryotic genes mapping approach and ciguatoxins (CTX)
