# Supplementary material for: Polyketide synthesis genes associated with toxin production in two species of Gambierdiscus (Dinophyceae)
Source: BMC Genomics. 2015 May 28;16(1):410. doi: 10.1186/s12864-015-1625-y (PMC4445524; doi:10.1186/s12864-015-1625-y)
Supplement: Additional file 1: Figure S1. — Correlation between cell dimensions (length of dorso-ventral axis of a cell) and quantity of nuclear DNA in 27 different species of dinoflagellates (23 genera). Figure S2. Phylogenetic analysis of H2A histone proteins. Table S1. Data used to estimate the correlation between cell dimensions and quantity of nuclear DNA in dinoflagellates (23 genera). Table S2. Description of different sequences from G. australes and G. belizeanus encoding essential enzymes for Glycolysis, TCA cycle, Oxidative phosphorylation, Carbon fixation (c3) cycle and pentose phosphate pathway. Table S3. A list of all the sequencing encoding the histone proteins found in the gene catalogue of G. australes and G. belizeanus. Table S4. A list of sequences from G. australes and G. belizeanus gene catalogue containing spliced leader at the 5’ end and polyA tail at the 3’ end. Table S5. Sequence properties of the transcripts encoding full ketoacyl synthase domain identified in G. australes CAWD149. Table S6. Sequence properties of the transcripts encoding full ketoacyl synthase domain identified in G. belizeanus. Table S7. Sequence properties of the transcripts encoding partial ketoacyl synthase domain identified in G. australes and G. belizeanus. Table S8. Sequence properties of the transcripts encoding full and partial ketoyl reductase domains identified in G. australes and G. belizeanus. Table S9. Sequence properties of the transcripts encoding full acyl carrier protein synthase, enoylreductase, acyltransferase, acyltransferase with ankyrin adaptor proteins, acyl carrier protein domains identified in G. australes and G. belizeanus. Table 10. Sequence properties of the transcripts encoding epoxidases, epoxide hydrolases and full and partial sulfotransferases enzymes identified in G. australes and G. belizeanus. Table S11. Sequence properties of the transcripts encoding full acyl carrier protein synthase, Enoyl reductase, acyl transferase, acyl carrier protein identified in G. australes and G. belize [file 12864_2015_1625_MOESM1_ESM.docx]

***Prorocentrum***

***Karenia***

***Alexandrium***

***Gambierdiscus***

**Other species**

Supplementary figure 1: Correlation between cell dimensions (length of dorso-ventral axis of a cell) and quantity of nuclear DNA in 27 different species of dinoflagellates (23 genera). The cell dimensions and the amount of nuclear DNA *Gambierdiscus australes* and *Gambierdiscus belizeanus* were calculated in this study. Other data were taken from [[1-3](#_ENREF_1)]. Four genera consisting of the largest amount of nuclear DNA (*Prorocentrum*, *Karenia*, *Alexandrium* and *Gambierdiscus*) are shown in colour. A linear trend line is included (r^2^=0.67, y=2.1038x – 1.4263). The correlation between cell dimensions and the amount of nuclear DNA in dinoflagellates is not linear, as predicted by earlier studies [[1](#_ENREF_1), [2](#_ENREF_2)].

Supplementary figure 2: Phylogenetic analysis of H2A histone proteins. Twenty-four sequences encoding H2A.X and H2A.Z variants of the H2A histone family representing 14 taxa were analysed by a maximum likelihood approach using the Le Gascuel substitution model and 100 bootstraps. H3 and H4 histones from *G. belizeanus* were used as an outgroup. Within the H2A clade H2A.X and H2A.Z variants from distinct well supported clades. Dinoflagellate H2A.X histones (coloured in red) form a well-supported clade within the H2A.X group.

Supplementary table 1: Data used to estimate the correlation between cell dimensions (length of dorso-ventral axis of a cell) and quantity of nuclear DNA in 27 different species of dinoflagellates (23 genera). The cell dimensions and the amount of nuclear DNA *Gambierdiscus australes* and *Gambierdiscus belizeanus* were calculated in this study. Other data were taken from Veldhuis et al., 1997, LaJeunesse et al., 2005 and Shoguchi et al., 2013.

| **Organism** | **Length of dorso-ventral axis of a cell** | **DNA content (pg cell^-1^)** |
| --- | --- | --- |
| *Alexandrium insuetum* | 25 | 30.8 |
| *Alexandrium lusitanicum* | 18 | 31 |
| *Alexandrium tamarense* | 25 | 103.5 |
| *Amphidinium carterae* | 15 | 5.9 |
| *Gymnodinium simplex* | 10 | 11.6 |
| *Heterocapsa triquetra* | 19 | 24.1 |
| *Heterocapsa pygmaea* | 11.5 | 3.8 |
| *Polarella flacialis* | 11.5 | 7 |
| *Karenia brevis* | 24 | 57.1 |
| *Karenia mikimotoi* | 29 | 100.1 |
| *Karlodinium galatheanum* | 14 | 16.9 |
| *Karlodinium galatheanum* | 14 | 16.3 |
| *Karlodinium rotundatum* | 13.5 | 3.6 |
| *Pfisteria piscicida* | 11 | 5.5 |
| *Pfisteria shumwayae* | 14 | 19.8 |
| *Prorocentrum balticum* | 12.5 | 8.3 |
| *Prorocentrum dentatum* | 15.5 | 6.6 |
| *Prorocentrum micans* | 30 | 115.2 |
| *Prorocentrum minimum* | 12.5 | 6.9 |
| *Symbiodinium* Clade A | 13.5 | 2.2 |
| *Symbiodinium* Clade B | 7.5 | 1.9 |
| *Symbiodinium* Clade C | 11 | 4.8 |
| *Symbiodinium* Clade D | 8.75 | 3.5 |
| *Symbiodinium* Clade F | 8.5 | 3 |
| *Gambierdiscus australes* | 38.7 | 33.2 |
| *Gambierdiscus belizeanus* | 48.1 | 35.8 |

Table 2: Description of different sequences from *G. australes* and *G. belizeanus* encoding essential enzymes for Glycolysis, TCA cycle, Oxidative phosphorylation, Carbon fixation (c3) cycle and pentose phosphate pathway.

| **Enzyme codes** | **Seq. Name** | **Blast Hit Description (HSP)** | **Length** | **Similarity (%)** |
| --- | --- | --- | --- | --- |
| **Glycolysis** | | | | |
| 2.7.1.2 Glucokinase | 149_50977 | gi\|299469720\|emb\|CBN76574.1\|Glucokinase [*Ectocarpus siliculosus*] | 1299 | 60 |
|  | 401_contig_8781 | gi\|530729626\|gb\|EQC27868.1\|glucokinase, variant [*Saprolegnia diclina* VS20] | 1319 | 60 |
| 5.3.1.9 Glucose-6-phosphate isomerase | 149_20260 | gi\|111609771\|gb\|ABH11438.1\|cytosolic glucose-6-phosphate isomerase [*Pyrocystis lunula*] | 1806 | 92 |
|  | 401_contig_7225 | gi\|111609771\|gb\|ABH11438.1\|cytosolic glucose-6-phosphate isomerase [*Pyrocystis lunula*] | 1830 | 92 |
| 2.7.1.11 6-phosphofructokinase | 149_6914 | gi\|298710116\|emb\|CBJ31829.1\| Short=PFP; AltName: Full=6-phosphofructokinase [*Ectocarpus siliculosus*] | 3887 | 65 |
|  | 401_contig_3477 | gi\|298710116\|emb\|CBJ31829.1\| Short=PFP; AltName: Full=6-phosphofructokinase [*Ectocarpus siliculosus*] | 4043 | 63 |
| 4.1.2.13 Fructose-bisphosphate aldolase | 149_709_sl | gi\|380715029\|gb\|AFE02909.1\|fructose-1,6-bisphosphate aldolase class II [*Emiliania huxleyi*] | 1540 | 87 |
|  | 401_contig_1721 | gi\|380715029\|gb\|AFE02909.1\|fructose-1,6-bisphosphate aldolase class II [*Emiliania huxleyi*] | 1612 | 86 |
| 5.3.1.1 Triose phosphate isomerase | 149_18489 | gi\|485606501\|gb\|EOD06277.1\|triose-phosphate isomerase [*Emiliania huxleyi* CCMP1516] | 1140 | 84 |
|  | 401_contig_10431_polyA | gi\|294877884\|ref\|XP_002768175.1\|triose-phosphate isomerase 1, putative [*Perkinsus marinus* ATCC 50983] | 918 | 73 |
| 1.2.1.12 Glyceraldehyde 3-phosphate dehydrogenase | 149_30784 | gi\|112253337\|gb\|ABI14256.1\|glyceraldehyde-3-phosphate dehydrogenase [*Pfiesteria piscicida*] | 2389 | 88 |
|  | 401_contig_22997 | gi\|134037044\|gb\|ABO47862.1\|glyceraldehyde-3-phosphate dehydrogenase [*Alexandrium fundyense*] | 929 | 96 |
| 2.7.2.3 Phosphoglycerate kinase | 149_2212 | gi\|58613475\|gb\|AAW79324.1\|phosphoglycerate kinase [*Heterocapsa triquetra*] | 1244 | 91 |
|  | 401_contig_47072 | gi\|428185881\|gb\|EKX54732.1\|hypothetical protein GUITHDRAFT_149840 [*Guillardia theta* CCMP2712] | 1103 | 80 |
| 5.4.2.1 Phosphoglycerate mutase | 149_8274_polyA | gi\|301110701\|ref\|XP_002904430.1\|2,3-bisphosphoglycerate-dependent phosphoglycerate mutase [*Phytophthora infestans* T30-4] | 1075 | 74 |
|  | 401_contig_35852 | gi\|301110701\|ref\|XP_002904430.1\|2,3-bisphosphoglycerate-dependent phosphoglycerate mutase [*Phytophthora infestans* T30-4] | 1100 | 73 |
| 4.2.1.11 Enolase | 149_13844_sl | gi\|40949688\|gb\|AAR97555.1\|enolase [*Heterocapsa triquetra*] | 1682 | 92 |
|  | 401_contig_8907_SL | gi\|40949688\|gb\|AAR97555.1\|enolase [*Heterocapsa triquetra*] | 1486 | 92 |
| 2.7.1.40 Pyruvate kinase | 149_13765 | gi\|294954240\|ref\|XP_002788069.1\|Pyruvate kinase, putative [*Perkinsus marinus* ATCC 50983] | 1742 | 75 |
|  | 401_contig_3463 | gi\|294954240\|ref\|XP_002788069.1\|Pyruvate kinase, putative [*Perkinsus marinus* ATCC 50983] | 1640 | 76 |
| **TCA Cycle** | | | | |
| 1.2.4.1/2.3.1.12: Pyruvate dehydrogenase E1 component/ Pyruvate dehydrogenase E2 component (dihydrolipoamide acetyltransferase) | 149_93716 | gi\|330845755\|ref\|XP_003294737.1\|pyruvate dehydrogenase E1 beta subunit [*Dictyostelium purpureum*] | 1187 | 82 |
|  | 149_68292 | gi\|66800847\|ref\|XP_629349.1\|pyruvate dehydrogenase E1 alpha subunit [*Dictyostelium discoideum* AX4] | 1187 | 78 |
|  | 149_15407 | gi\|516925236\|ref\|WP_018169041.1\|pyruvate dehydrogenase [*Thioalkalivibrio* sp. ALMg9] | 3368 | 67 |
|  | 401_contig_39936 | gi\|516925236\|ref\|WP_018169041.1\|pyruvate dehydrogenase [*Thioalkalivibrio* sp.] | 3291 | 66 |
| 2.3.3.1: Citrate synthase | 149_32793 | gi\|319997130\|gb\|ADV91159.1\|mitochondrial citrate synthase-like protein 1 [*Karlodinium micrum*] | 1658 | 84 |
|  | 401_contig_11876 | gi\|319997130\|gb\|ADV91159.1\|mitochondrial citrate synthase-like protein 1 [*Karlodinium veneficum*] | 1558 | 83 |
|  | 149_36492 | gi\|294932837\|ref\|XP_002780466.1\|2-methylcitrate synthase, putative [*Perkinsus marinus* ATCC 50983] | 1803 | 77 |
|  | 401_contig_4604 | gi\|294932837\|ref\|XP_002780466.1\|2-methylcitrate synthase, putative [*Perkinsus marinus* ATCC 50983] | 1626 | 75 |
| 4.2.1.3: Aconitate hydratase | 149_62279 | gi\|449493135\|ref\|XP_004159202.1\|PREDICTED: aconitate hydratase, cytoplasmic-like [*Cucumis sativus*] | 2791 | 71 |
|  | 401_contig_50030 | gi\|868003\|dbj\|BAA06108.1\| aconitase [*Cucurbita cv. Kurokawa Amakuri*] | 2818 | 71 |
|  | 149_1001_polyA | gi\|255074481\|ref\|XP_002500915.1\|predicted protein [*Micromonas* sp. RCC299] | 2950 | 82 |
|  | 401_contig_9990_polyA | gi\|255074481\|ref\|XP_002500915.1\|predicted protein [*Micromonas* sp. RCC299] | 2927 | 82 |
| 1.1.1.42: Isocitrate dehydrogenase | 149_6477 | gi\|319997134\|gb\|ADV91161.1\|mitochondrial isocitrate dehydrogenase, partial [*Karlodinium micrum*] | 2156 | 71 |
|  | 401_contig_7512 | gi\|319997134\|gb\|ADV91161.1\|mitochondrial isocitrate dehydrogenase, partial [*Karlodinium veneficum*] | 2207 | 71 |
| 1.2.4.2/2.3.1.61: 2-oxoglutarate dehydrogenase E1 component / 2-oxoglutarate dehydrogenase E2 component (dihydrolipoamide succinyltransferase) | 149_28319 | gi\|224009017\|ref\|XP_002293467.1\|2-oxoglutarate dehydrogenase E1 component [*Thalassiosira pseudonana* CCMP1335] | 3268 | 66 |
|  | 401_contig_38175 | gi\|219123641\|ref\|XP_002182131.1\|2-oxoglutarate dehydrogenase E1 component [*Phaeodactylum tricornutum* CCAP 1055/1] | 2906 | 69 |
|  | 149_1372 | gi\|301097778\|ref\|XP_002897983.1\|dihydrolipoamide succinyltransferase, putative [*Phytophthora infestans* T30-4] | 1698 | 70 |
|  | 401_contig_18205 | gi\|470431753\|ref\|XP_004338432.1\|hypothetical protein ACA1_320830 [*Acanthamoeba castellanii* str. Neff] | 1537 | 70 |
| 6.2.1.4/6.2.1.5: Succinyl-CoA synthetase alpha subunit | 149_1608 | gi\|300176246\|emb\|CBK23557.2\|Succinyl-CoA Synthetase subunit [*Blastocystis hominis*] | 1153 | 83 |
|  | 401_contig_5044 | gi\|158634608\|gb\|ABW76150.1\|succinyl-CoA synthetase alpha subunit [*Blastocystis* sp. NandII] | 1261 | 84 |
| 1.3.5.1/ 1.3.99.1: Succinate dehydrogenase (ubiquinone) flavoprotein subunit/ Succinate dehydrogenase flavoprotein subunit | 149_11334_polyA | 1.3.5.1-gi\|319997162\|gb\|ADV91175.1\|mitochondrial succinate dehydrogenase flavoprotein-like protein 1, partial [*Karlodinium micrum*] | 1420 | 92 |
|  | 401_contig_15784 | gi\|294945512\|ref\|XP_002784717.1\|conserved hypothetical protein [*Perkinsus marinus* ATCC 50983] | 2071 | 87 |
|  | 149_12355 | 1.3.99.1- gi\|294904393\|ref\|XP_002777585.1\|succinate dehydrogenase, putative [*Perkinsus marinus* ATCC 50983] | 2336 | 72 |
|  | 401_contig_2501 | gi\|58613585\|gb\|AAW79379.1\|probable fumerate reductase [*Heterocapsa triquetra*] | 1767 | 87 |
| 4.2.1.2: Fumarate hydratase, class I | 149_10526_polyA | gi\|326431282\|gb\|EGD76852.1\|fumarate hydratase class I [*Salpingoeca* sp. ATCC 50818] | 1945 | 77 |
|  | 401_contig_1647 | gi\|514687294\|ref\|XP_004991224.1\|fumarate hydratase class I [*Salpingoeca* sp. ATCC 50818] | 2160 | 79 |
| 1.1.1.37: Malate dehydrogenase | 149_9118_polyA | gi\|58613463\|gb\|AAW79318.1\|malate dehydrogenase [*Heterocapsa triquetra*] | 1668 | 90 |
|  | 401_contig_174_polyA | gi\|58613463\|gb\|AAW79318.1\|malate dehydrogenase [*Heterocapsa triquetra*] | 1740 | 89 |
| **Oxidative phosphorylation** | | | | |
| Complex I |  |  |  |  |
| 1.6.5.3 NADH dehydrogenase | 149_76902_polyA | \|XP_003448509.1\|PREDICTED: NADH dehydrogenase [ubiquinone] flavoprotein 2, mitochondrial-like isoform 3 [*Oreochromis niloticu*] | 874 | 75 |
| Complex II |  |  |  |  |
| 1.3.5.1 Succinate dehydrogenase flavoprotein subunit | 149_88596_polyA | gi\|195647178\|gb\|ACG43057.1\|succinate dehydrogenase flavoprotein subunit,mitochondrial precursor [*Zea mays*] | 1973 | 80 |
| 1.3.99.1 Succinate dehydrogenase flavoprotein subunit | 149_12355 | gi\|294904393\|ref\|XP_002777585.1\|succinate dehydrogenase, putative [*Perkinsus marinus* ATCC 50983] | 2336 | 72 |
|  | 401_contig_2501 | gi\|58613585\|gb\|AAW79379.1\|probable fumerate reductase [*Heterocapsa triquetra*] | 1767 | 87 |
| Complex III |  |  |  |  |
| 1.10.2.2 Ubiquinol-cytochrome c reductase iron sulfur subunit | 149_3123 | gi\|401412972\|ref\|XP_003885933.1\|Ubiquinol-cytochrome c reductase cytochrome c1 subunit, related [*Neospora caninum* Liverpool] | 1174 | 82 |
|  | 401_contig_7321 | gi\|387762379\|dbj\|BAM15621.1\|cytochrome c1 precursor [*Plasmodium gallinaceum*] | 1319 | 80 |
| Complex IV |  |  |  |  |
| 1.9.3.1 Cb-type cytochrome c oxidase subunit I | 149_10335_sl_polyA | gi\|239870418\|gb\|EER00939.1\| Cg8 protein, putative [*Perkinsus marinus* ATCC 50983] | 866 | 76 |
|  | 401_contig_44353_SL | gi\|294877976\|ref\|XP_002768221.1\|Cg8 protein, putative [*Perkinsus marinus* ATCC 50983] | 782 | 75 |
|  | 149_2285 | gi\|221059717\|ref\|XP_002260504.1\|cytochrome c oxidase assembly protein [*Plasmodium knowlesi* strain H] | 1197 | 74 |
|  | 401_contig_13776_polyA | gi\|294953231\|ref\|XP_002787660.1\|hypothetical protein, conserved [*Perkinsus marinus* ATCC 50983] | 1441 | 73 |
| Complex V |  |  |  |  |
| 3.6.3.6 H+ transporting ATPase | 149_11648_polyA | gi\|422293434\|gb\|EKU20734.1\|H+-transporting ATPase [*Nannochloropsis gaditana* CCMP526 | 3223 | 70 |
|  | 401_contig_524_polyA | gi\|224004642\|ref\|XP_002295972.1\|predicted protein [*Thalassiosira pseudonana* CCMP1335] | 3047 | 69 |
| 3.6.3.10 H+/K+-exchanging ATPase | 149_8793 | gi\|296481783\|tpg\|DAA23898.1\| TPA: non-gastric H+,K+-ATPase-like [*Bos taurus*] | 3759 | 50 |
|  | 401_contig_2910 | gi\|470241726\|ref\|XP_004352438.1\|P-type ATPase [*Dictyostelium fasciculatum*] | 3673 | 51 |
| **Carbon fixation (C3) pathway** | | | | |
| 2.7.1.19 Phosphoribulokinase | 149_2313_sl | gi\|60101676\|gb\|AAX13962.1\|chloroplast phosphoribulokinase [*Pyrocystis lunula*] | 1553 | 95 |
|  | 401_contig_13969 | gi\|60101676\|gb\|AAX13962.1\|chloroplast phosphoribulokinase [*Pyrocystis lunula*] | 1197 | 95 |
| 4.1.1.39 Ribulose bisphosphate carboxylase large chain | 149_2636 | gi\|75282236\|sp\|Q41406.1\|RBL2_SYMSPRecName: Full=Ribulose bisphosphate carboxylase; [*Symbiodinium* sp.] | 4768 | 92 |
|  | 401_contig_16535 | gi\|84029424\|sp\|Q42813.2\|RBL2_GONPORecName: Full=Ribulose 1,5-bisphosphate carboxylase, chloroplastic; Flags: Precursor | 1037 | 92 |
| 2.7.2.3 Phosphoglycerate kinase | 149_2212 | gi\|58613475\|gb\|AAW79324.1\|phosphoglycerate kinase [*Heterocapsa triquetra*] | 1244 | 91 |
|  | 401_contig_47072 | gi\|428185881\|gb\|EKX54732.1\|hypothetical protein GUITHDRAFT_149840 [*Guillardia theta* CCMP2712] | 1103 | 80 |
| 1.2.1.13 Glyceraldehyde -3- phosphate dehydrogenase (NADP+) | 149_5701 | glyceraldehyde-3-phosphate dehydrogenase isoform 2 [*Lingulodinium polyedrum*]. | 1397 | 79 |
|  | 401_contig_1796 | gi\|4103871\|gb\|AAD01870.1\|glyceraldehyde-3-phosphate dehydrogenase [*Lingulodinium polyedrum*] | 1636 | 84 |
| 5.3.1.1 Triosephosphate isomerase | 149_2730_polyA | gi\|157093165\|gb\|ABV22237.1\|triose-phosphate isomerase [*Karlodinium micrum*] | 1210 | 74 |
|  | 401_contig_1242 | gi\|485606501\|gb\|EOD06277.1\|triose-phosphate isomerase [*Emiliania huxleyi* CCMP1516] | 762 | 76 |
| 4.1.2.13 Fructose bisphosphate aldolase, class I | 149_709_SL | gi\|380715029\|gb\|AFE02909.1\|fructose-1,6-bisphosphate aldolase [*Emiliania huxleyi*] | 1540 | 87 |
|  | 401_contig_1721 | gi\|380715029\|gb\|AFE02909.1\|fructose-1,6-bisphosphate aldolase class II [*Emiliania huxleyi*] | 1612 | 86 |
| 3.1.3.11 Fructose-1,6-bisphospatase I | 149_5088_sl | gi\|219128059\|ref\|XP_002184240.1\|fructose-1,6-bisphosphatase [*Phaeodactylum tricornutum* CCAP 1055/1] | 1194 | 78 |
|  | 401_contig_938_polyA | gi\|323449412\|gb\|EGB05300.1\|hypothetical protein AURANDRAFT_31061 [*Aureococcus anophagefferens*] | 1453 | 78 |
|  | 149_5886 | gi\|99904124\|gb\|ABF68596.1\|chloroplast fructose-1,6-bisphosphatase [*Lingulodinium polyedrum*] | 1749 | 81 |
|  | 401_contig_36482 | gi\|99904124\|gb\|ABF68596.1\|chloroplast fructose-1,6-bisphosphatase [*Lingulodinium polyedrum*] | 1424 | 82 |
| 2.2.1.1 Transketolase | 149_48395_polyA | gi\|145105752\|gb\|ABP35605.1\|transketolase [*Karlodinium micrum*] | 2373 | 79 |
|  | 401_contig_14372 | gi\|145105752\|gb\|ABP35605.1\|transketolase [*Karlodinium veneficum*] | 2411 | 79 |
| 3.1.3.37 Sedoheptulose-1,7-bisphosphatase | 149_4019_polyA | gi\|99903657\|gb\|ABF68590.1\|chloroplast sedoheptulose-1,7-bisphosphatase [*Lingulodinium polyedrum*] | 1430 | 97 |
|  | 401_contig_14819_polyA | gi\|99903657\|gb\|ABF68590.1\|chloroplast sedoheptulose-1,7-bisphosphatase [*Lingulodinium polyedrum*] | 1457 | 96 |
| 5.1.3.1 Ribuloase-phosphate 3-epimerase | 149_43582_polyA | gi\|325179807\|emb\|CCA14210.1\|ribulosephosphate 3epimerase putative [*Albugo laibachii* Nc14] | 1090 | 70 |
|  | 401_contig_15107_polyA | gi\|325179807\|emb\|CCA14210.1\|ribulosephosphate 3epimerase putative [*Albugo laibachii* Nc14] | 1085 | 69 |
| 5.3.1.6 Ribose 5-phosphate isomerase A | 149_20187 | gi\|58613535\|gb\|AAW79354.1\|chloroplast ribose-5-phosphate isomerase [*Heterocapsa triquetra*] | 2239 | 84 |
|  | 401_contig_20190 | gi\|58613535\|gb\|AAW79354.1\|chloroplast ribose-5-phosphate isomerase [*Heterocapsa triquetra*] | 1437 | 94 |
| **Pentose phosphate pathway** | | | | |
| 1.1.1.49 glucose-6-phosphate dehydrogenase | 401_contig_32220 | Glucose-6-phosphate 1-dehydrogenase, related [*Neospora caninum* Liverpool] | 2633 | 55 |
| 3.1.1.31 6-phosphogluconolactonase | 149_8620 | [*Coccomyxa subellipsoidea* C-169] EIE21413.1 | 1039 | 59 |
|  | 401_68001_sl-polyA | hypothetical protein EMIHUDRAFT_440947 [*Emiliania huxleyi* CCMP1516]EOD35668.1 | 1502 | 50 |
| 1.1.1.44 phosphogluconate dehydrogenase (NADP+-dependent, decarboxylating) | 401_20476 | (XP_808031) 6-phosphogluconate dehydrogenase, decarboxylating [*Trypanosoma cruzi* strain CL Brener] | 1215 | 71 |
|  | 149_contig_25335_polyA | (CCC50754)putative 6-phosphogluconate dehydrogenase,decarboxylating [*Trypanosoma vivax* Y486] | 1616 | 68 |
| 5.3.1.6 ribose-5-phosphate isomerase | 401_20190 | chloroplast ribose-5-phosphate isomerase [*Heterocapsa triquetra*] (AAW79354) | 1437 | 94 |
|  | 149_contig_4371 | chloroplast ribose-5-phosphate isomerase [*Heterocapsa triquetra*] (AAW79354) | 1546 | 94 |
| 5.1.3.1 ribulose-phosphate 3-epimerase | 401_15107_polyA | gi\|325179807\|emb\|CCA14210.1\|ribulosephosphate 3epimerase putative [*Albugo laibachii* Nc14] | 1085 | 69 |
|  | 149_contig_43582_polyA | gi\|325179807\|emb\|CCA14210.1\|ribulosephosphate 3epimerase putative [*Albugo laibachii* Nc14] | 1090 | 70 |
| 2.2.1.1 transketolase | 401_8107 | gi\|440804204\|gb\|ELR25081.1\|transketolase [*Acanthamoeba castellanii* str. Neff] | 2943 | 59 |
|  | 149_contig_31292 | gi\|493032223\|ref\|WP_006101618.1\|transketolase [*Coleofasciculus chthonoplastes*] | 3064 | 48 |
| 2.2.1.2 transaldolase | 401_10755 | gi\|219130171\|ref\|XP_002185245.1\|plastidic transaldolase [*Phaeodactylum tricornutum* CCAP 1055/1] | 1358 | 81 |
|  | 149_contig_15069 | gi\|428168670\|gb\|EKX37612.1\|hypothetical protein GUITHDRAFT_77986 [*Guillardia theta* CCMP2712] | 1375 | 82 |

Supplementary table 3: A list of all the sequencing encoding the histone proteins (H2A, H2B, H3, H4) found in the gene catalogue of *G. australes* (sequence names starting with 149) and *G. belizeanus* (Sequence names starting with 401).

| **Seq. Name** | **Seq. Length** | **Blast Hit Description (HSP)** | **Similarity (%)** | **Query Frame** |
| --- | --- | --- | --- | --- |
| **H2A** | | | | |
| 149_contig_15788 | 732 | gi\|61658009\|gb\|AAX49407.1\|histone H2A.X [*Alexandrium tamarense*] | 93 | 1 |
| 149_contig_35634 | 712 | gi\|61658009\|gb\|AAX49407.1\|histone H2A.X [*Alexandrium tamarense*] | 83 | -3 |
| 401_contig_11026 | 705 | gi\|61658009\|gb\|AAX49407.1\|histone H2A.X [*Alexandrium tamarense*] | 82 | 2 |
| 401_contig_36597 | 726 | gi\|61658009\|gb\|AAX49407.1\|histone H2A.X [*Alexandrium tamarense*] | 82 | 3 |
| **H2B** | | | | |
| 149_contig_19166_polyA | 1035 | gi\|219116831\|ref\|XP_002179210.1\|histone H2B isoform 1b [*Phaeodactylum tricornutum* CCAP 1055/1] | 89 | -2 |
| 149_contig_47444 | 520 | gi\|513027284\|ref\|XP_004874791.1\|PREDICTED: histone H2B type 3-B-like [*Heterocephalus glaber*] | 78 | -3 |
| 401_contig_18580 | 844 | gi\|66362766\|ref\|XP_628349.1\|histone H2B [*Cryptosporidium parvum* Iowa II] | 87 | 3 |
| 401_contig_29169 | 494 | gi\|350641162\|gb\|AEQ34971.1\|histone h2B [*Tigriopus japonicus*] | 81 | 2 |
| 401_contig_33038 | 723 | gi\|512970936\|ref\|XP_004847604.1\|PREDICTED: histone H2B type 1-M-like [*Heterocephalus glaber*] | 74 | 1 |
| **H3** | | | | |
| 149_contig_9307_polyA | 873 | gi\|19880141\|gb\|AAM00267.1\|AF361949_1histone 3 [*Eimeria tenella*] | 95 | 3 |
| 149_contig_11254 | 568 | gi\|470321716\|ref\|XP_004349092.1\|histone t [*Capsaspora owczarzaki* ATCC 30864] | 64 | 2 |
| 149_contig_12715 | 417 | gi\|294943039\|ref\|XP_002783743.1\|histone H3, putative [*Perkinsus marinus* ATCC 50983] | 96 | -2 |
| 149_contig_27862_polyA | 562 | gi\|170053459\|ref\|XP_001862683.1\|histone H3.1t [*Culex quinquefasciatus*] | 95 | 3 |
| 149_contig_30604_polyA | 776 | gi\|9369379\|gb\|AAF87128.1\|AC006434_24F10A5.19 [*Arabidopsis thaliana*] | 55 | -3 |
| 149_contig_38662 | 770 | gi\|32346218\|gb\|AAN85430.1\|histone H3 [*Pyrocystis lunula*] | 71 | 1 |
| 149_contig_66978 | 423 | gi\|294878494\|ref\|XP_002768392.1\|histone H3, putative [*Perkinsus marinus* ATCC 50983] | 96 | 1 |
| 401_contig_593 | 678 | gi\|19880141\|gb\|AAM00267.1\|AF361949_1histone 3 [*Eimeria tenella*] | 95 | 2 |
| 401_contig_9159 | 698 | gi\|312087725\|ref\|XP_003145584.1\|histone type 2 [*Loa loa*] | 62 | -2 |
| 401_contig_11824 | 403 | gi\|397571476\|gb\|EJK47817.1\|hypothetical protein THAOC_33441, partial [*Thalassiosira oceanica*] | 74 | -1 |
| 401_contig_20196 | 677 | gi\|32346218\|gb\|AAN85430.1\|histone H3 [*Pyrocystis lunula*] | 70 | -1 |
| 401_contig_34651 | 447 | gi\|109148993\|ref\|XP_001083065.1\|PREDICTED: histone H3.3 type 2-like isoform 1 [*Macaca mulatta*] | 92 | 1 |
| 401_contig_35160 | 541 | gi\|109148993\|ref\|XP_001083065.1\|PREDICTED: histone H3.3 type 2-like isoform 1 [*Macaca mulatta* | 92 | -1 |
| **H4** | | | | |
| 149_contig_2757 | 880 | gi\|50548501\|ref\|XP_501720.1\|YALI0C11385p [*Yarrowia lipolytica*] | 70 | -1 |
| 149_contig_14443 | 567 | gi\|543748774\|ref\|XP_005515123.1\|PREDICTED: histone H4-like [*Columba livia*] | 94 | 3 |
| 149_contig_14444 | 737 | gi\|388579081\|gb\|EIM19410.1\|histone 4, partial [*Wallemia sebi* CBS 633.66] | 93 | 2 |
| 401_contig_9743 | 525 | gi\|543748774\|ref\|XP_005515123.1\|PREDICTED: histone H4-like [*Columba livia*] | 94 | 3 |
| 401_contig_28185 | 577 | gi\|543748774\|ref\|XP_005515123.1\|PREDICTED: histone H4-like [*Columba livia*] | 94 | 2 |

Supplementary table 4: A list of sequences from *G. australes* (sequences starting with 149) and *G. belizeanus* (sequences starting with 401) gene catalogue containing spliced leader at the 5’ end and polyA tail at the 3’ end. Only sequences with assigned enzyme codes were included in this table.

| **Seq. Name** | **Seq. Length** | **Blast Hit Description (HSP)** | **Similarity (%)** | **Enzyme codes** |
| --- | --- | --- | --- | --- |
| 149_contig_487_sl_polyA | 1167 | gi\|255965613\|gb\|ACU45109.1\|unknown [*Pfiesteria piscicida*] | 72 | EC:2.1.1.36 |
| 149_contig_792_sl_polyA | 1718 | gi\|221482892\|gb\|EEE21223.1\|glycylpeptide N-tetRadecanoyltransferase, putative [*Toxoplasma gondii* GT1] | 75 | EC:2.3.1.97 |
| 149_contig_1184_sl_polyA | 1880 | gi\|340503236\|gb\|EGR29846.1\|protein kinase domain protein [*Ichthyophthirius multifiliis*] | 73 | EC:3.1.1.3; EC:2.7.10.0; EC:2.7.11.25; EC:3.1.1.21 |
| 149_contig_1233_sl_polyA | 2157 | gi\|294891367\|ref\|XP_002773544.1\|tonneau, putative [*Perkinsus marinus* ATCC 50983] | 61 | EC:2.7.10.0 |
| 149_contig_2658_sl_polyA | 1687 | gi\|340708105\|pdb\|3RP9\|AChain A, Crystal Structure Of The Apo Mapk From *Toxoplasma gondii*, 25.M01780 Or Tgme49_007820 | 57 | EC:2.7.11.24 |
| 149_contig_3689_sl_polyA | 2825 | gi\|290977828\|ref\|XP_002671639.1\|hypothetical protein NAEGRDRAFT_59412 [*Naegleria gruberi*] | 44 | EC:6.3.2.19 |
| 149_contig_4127_sl_polyA | 1067 | gi\|168056614\|ref\|XP_001780314.1\|predicted protein [*Physcomitrella patens* subsp. patens] | 55 | EC:6.3.2.19 |
| 149_contig_4933_sl_polyA | 2218 | gi\|397905848\|ref\|ZP_10506686.1\|Glucosylceramidase [*Caloramator australicus* RC3] | 58 | EC:3.2.1.45 |
| 149_contig_5374_sl_polyA | 2798 | gi\|308814306\|ref\|XP_003084458.1\|tesmin/TSO1-like CXC domain-containing protein (ISS) [*Ostreococcus tauri*] | 42 | EC:6.3.2.19 |
| 149_contig_5376_sl_polyA | 1123 | gi\|301115680\|ref\|XP_002905569.1\|conserved hypothetical protein [*Phytophthora infestans* T30-4] | 64 | EC:6.3.2.0 |
| 149_contig_5997_sl_polyA | 2952 | gi\|294891359\|ref\|XP_002773540.1\|phosphatidylinositol-4-phosphate 5-kinase, putative [*Perkinsus marinus* ATCC 50983] | 73 | EC:1.6.3.1; EC:2.7.1.68 |
| 149_contig_6074_sl_polyA | 1037 | gi\|302762348\|ref\|XP_002964596.1\|hypothetical protein SELMODRAFT_81184 [*Selaginella moellendorffii*] | 66 | EC:1.3.3.4; EC:1.4.3.5 |
| 149_contig_7239_sl_polyA | 1658 | gi\|262195977\|ref\|YP_003267186.1\|coproporphyrinogen III oxidase [*Haliangium ochraceum* DSM 14365] | 62 | EC:1.3.3.3 |
| 149_contig_8943_sl_polyA | 955 | gi\|134117962\|ref\|XP_772362.1\|hypothetical protein CNBL2300 [*Cryptococcus neoformans* var. neoformans B-3501A] | 66 | EC:4.2.1.109 |
| 149_contig_9363_sl_polyA | 1417 | gi\|255083911\|ref\|XP_002508530.1\|histone deacetylase, SIR2 family [*Micromonas* sp. RCC299] | 67 | EC:3.5.1.0 |
| 149_contig_10335_sl_polyA | 866 | gi\|294877976\|ref\|XP_002768221.1\|Cg8 protein, putative [*Perkinsus marinus* ATCC 50983] | 76 | EC:1.9.3.1 |
| 149_contig_11272_sl_polyA | 2161 | gi\|295923842\|gb\|ADG63074.1\|cellulase [*Lingulodinium polyedrum*] | 60 | EC:3.2.1.91 |
| 149_contig_11632_sl_polyA | 1609 | gi\|301099961\|ref\|XP_002899071.1\|histone arginine demethylase, putative [*Phytophthora infestans* T30-4] | 53 | EC:1.13.11.0; EC:2.1.1.0 |
| 149_contig_12119_sl_polyA | 1532 | gi\|348666130\|gb\|EGZ05958.1\|hypothetical protein PHYSODRAFT_356188 [*Phytophthora sojae*] | 56 | EC:6.3.2.0 |
| 149_contig_12564_sl_polyA | 2190 | gi\|294893708\|ref\|XP_002774607.1\|calcium-dependent protein kinase, putative [*Perkinsus marinus* ATCC 50983] | 65 | EC:1.6.3.1; EC:2.7.11.17 |
| 149_contig_12675_sl_polyA | 1339 | gi\|2289782\|dbj\|BAA21673.1\|cdc2 kinase [*Allium cepa*] | 63 | EC:2.7.11.22; EC:2.7.11.23 |
| 149_contig_13376_sl_polyA | 1128 | gi\|294054648\|ref\|YP_003548306.1\|FKBP-type peptidylprolyl isomerase [*Coraliomargarita akajimensis* DSM 45221] | 63 | EC:5.2.1.8 |
| 149_contig_13699_sl_polyA | 2127 | gi\|320528215\|ref\|ZP_08029379.1\|conserved domain protein [*Solobacterium moorei* F0204] | 65 | EC:3.1.3.48 |
| 149_contig_14058_sl_polyA | 1776 | gi\|302756173\|ref\|XP_002961510.1\|hypothetical protein SELMODRAFT_63506 [*Selaginella moellendorffii*] | 42 | EC:3.6.1.6 |
| 149_contig_15154_sl_polyA | 1083 | gi\|433646875\|ref\|YP_007291877.1\|enoyl-CoA hydratase/carnithine racemase [*Mycobacterium smegmatis* JS623] | 59 | EC:4.2.1.17 |
| 149_contig_15695_sl_polyA | 1491 | gi\|290971738\|ref\|XP_002668638.1\|predicted protein [*Naegleria gruberi*] >gi\|284082120\|gb\|EFC35894.1\| predicted protein [*Naegleria gruberi*] | 57 | EC:2.7.11.0 |
| 149_contig_15730_sl_polyA | 1499 | gi\|294955794\|ref\|XP_002788683.1\|mitogen-activated protein kinase 2, putative [*Perkinsus marinus* ATCC 50983] | 77 | EC:2.7.11.24 |
| 149_contig_16291_sl_polyA | 1556 | gi\|301104296\|ref\|XP_002901233.1\|conserved hypothetical protein [*Phytophthora infestans* T30-4] | 52 | EC:1.14.11.0 |
| 149_contig_16433_sl_polyA | 3549 | gi\|159472657\|ref\|XP_001694461.1\|predicted protein [*Chlamydomonas reinhardtii*] | 61 | EC:1.5.1.23; EC:1.5.1.17; EC:1.5.1.11; EC:5.4.2.1; EC:1.1.1.0; EC:1.5.1.22 |
| 149_contig_16831_sl_polyA | 1499 | gi\|340503679\|gb\|EGR30218.1\|protein kinase domain protein [*Ichthyophthirius multifiliis*] | 72 | EC:2.7.10.0; EC:2.7.11.24 |
| 149_contig_17788_sl_polyA | 2117 | gi\|398345192\|ref\|ZP_10529895.1\|cellulase [*Leptospira inadai* serovar Lyme str. 10] | 53 | EC:3.2.1.0 |
| 149_contig_18729_sl_polyA | 471 | gi\|258549157\|ref\|XP_002585482.1\|conserved Plasmodium protein [*Plasmodium falciparum* 3D7] | 79 | EC:2.7.4.8 |
| 149_contig_21077_sl_polyA | 1550 | gi\|50547503\|ref\|XP_501221.1\|YALI0B22440p [*Yarrowia lipolytica*] | 66 | EC:2.5.1.54 |
| 149_contig_21492_sl_polyA | 1998 | gi\|294953595\|ref\|XP_002787842.1\|fk506 binding protein, putative [*Perkinsus marinus* ATCC 50983] | 63 | EC:5.2.1.8 |
| 149_contig_21629_sl_polyA | 810 | gi\|112253395\|gb\|ABI14285.1\|cyclophilin-like protein [*Pfiesteria piscicida*] | 91 | EC:5.2.1.8 |
| 149_contig_23573_sl_polyA | 1210 | gi\|294932215\|ref\|XP_002780161.1\|hypothetical protein Pmar_PMAR019058 [*Perkinsus marinus* ATCC 50983] | 43 | EC:3.1.4.0 |
| 149_contig_24223_sl_polyA | 1471 | gi\|119474724\|ref\|ZP_01615077.1\|acetyl-CoA acetyltransferase [marine gamma proteobacterium HTCC2143] | 73 | EC:2.3.1.9 |
| 149_contig_24497_sl_polyA | 2880 | gi\|403375863\|gb\|EJY87907.1\|Protein kinase domain containing protein [*Oxytricha trifallax*] | 79 | EC:3.1.13.4; EC:3.1.1.4; EC:3.1.1.47; EC:2.7.11.14; EC:2.7.11.13; EC:2.7.11.11 |
| 149_contig_27600_sl_polyA | 1290 | gi\|357464999\|ref\|XP_003602781.1\|RNA pseudourine synthase [*Medicago truncatula*] | 44 | EC:5.4.99.12 |
| 149_contig_30975_sl_polyA | 2032 | gi\|428185941\|gb\|EKX54792.1\|hypothetical protein GUITHDRAFT_159096 [*Guillardia theta* CCMP2712] | 65 | EC:3.4.13.0 |
| 149_contig_35014_sl_polyA | 2522 | gi\|116206484\|ref\|XP_001229051.1\|hypothetical protein CHGG_02535 [*Chaetomium globosum* CBS 148.51] | 42 | EC:5.3.4.1; EC:2.3.2.13 |
| 149_contig_40810_sl_polyA | 1287 | gi\|397597691\|gb\|EJK57063.1\|hypothetical protein THAOC_22935 [*Thalassiosira oceanica*] | 64 | EC:3.4.23.0 |
| 149_contig_41177_sl_polyA | 1645 | gi\|146197065\|dbj\|BAF57296.1\|putative glycosyl hydrolase family7 [uncultured symbiotic protist of Reticulitermes speratus] | 42 | EC:3.2.1.0 |
| 149_contig_41772_sl_polyA | 782 | gi\|290994392\|ref\|XP_002679816.1\|predicted protein [*Naegleria gruberi*] | 64 | EC:2.7.11.0 |
| 149_contig_42238_sl_polyA | 972 | gi\|123438216\|ref\|XP_001309895.1\|Bromodomain containing protein [*Trichomonas vaginalis* G3] | 56 | EC:2.3.1.48 |
| 149_contig_45844_sl_polyA | 972 | gi\|386812653\|ref\|ZP_10099878.1\|pyrroline-5-carboxylate reductase [planctomycete KSU-1] | 67 | EC:1.5.1.2 |
| 149_contig_57196_sl_polyA | 1375 | gi\|225164568\|ref\|ZP_03726817.1\|hypothetical protein ObacDRAFT_6144 [*Diplosphaera colitermitum* TAV2] | 46 | EC:2.1.1.0 |
| 401_contig_447__SL-polyA | 1889 | gi\|255087216\|ref\|XP_002505531.1\|predicted protein [*Micromonas* sp. RCC299] | 68 | EC:3.4.16.0 |
| 401_contig_731_SL-polyA | 920 | gi\|449455401\|ref\|XP_004145441.1\|PREDICTED: proteasome subunit alpha type-2-B-like [*Cucumis sativus*] | 88 | EC:3.4.25.0 |
| 401_contig_10838__SL-polyA | 1215 | gi\|397630719\|gb\|EJK69883.1\|hypothetical protein THAOC_08818 [*Thalassiosira oceanica*] | 54 | EC:2.7.7.0 |
| 401_contig_17392__SL-polyA | 3896 | gi\|414091051\|gb\|AFW98413.1\|type I polyketide synthase [*Alexandrium ostenfeldii*] | 47 | EC:2.3.1.0 |
| 401_contig_23986__SL-polyA | 1461 | gi\|348678419\|gb\|EGZ18236.1\|hypothetical protein PHYSODRAFT_498283 [*Phytophthora sojae*] | 75 | EC:2.4.2.0 |
| 401_contig_25603_SL-polyA | 2561 | gi\|364284970\|gb\|AEW47962.1\|GHF1 protein [uncultured bacterium F2_16] | 57 | EC:3.2.1.38; EC:3.2.1.39; EC:3.2.1.23 |
| 401_contig_30852__SL-polyA | 2096 | gi\|294946092\|ref\|XP_002784924.1\|conserved hypothetical protein [*Perkinsus marinus* ATCC 50983] | 60 | EC:3.4.22.0 |
| 401_contig_30895_SL-polyA | 870 | gi\|294948698\|ref\|XP_002785850.1\|Charged multivesicular body protein, putative [*Perkinsus marinus* ATCC 50983] | 61 | EC:3.4.25.0 |
| 401_contig_32833_SL-polyA | 2302 | gi\|66357412\|ref\|XP_625884.1\|Dbp1p, eIF4a-1 family RNA SFII helicase (DEXDC+HELICc) [*Cryptosporidium parvum* Iowa II] | 65 | EC:3.6.1.15 |
| 401_contig_35644_SL-polyA | 1063 | gi\|145525607\|ref\|XP_001448620.1\|hypothetical protein [*Paramecium tetraurelia* strain d4-2] | 58 | EC:2.1.1.0 |
| 401_contig_36277_SL-polyA | 1274 | gi\|375149726\|ref\|YP_005012167.1\|FkbM family methyltransferase [*Niastella koreensis* GR20-10] | 50 | EC:2.1.1.0 |
| 401_contig_36304__SL-polyA | 431 | gi\|294878985\|ref\|XP_002768537.1\|Casein kinase I, putative [*Perkinsus marinus* ATCC 50983] | 83 | EC:2.7.11.0 |
| 401_contig_58360_SL-polyA | 2050 | gi\|119944895\|ref\|YP_942575.1\|S-adenosyl-methyltransferase MraW [*Psychromonas ingrahamii* 37] | 56 | EC:2.1.1.0 |

Supplementary table 5: Sequence properties of the transcripts encoding full ketoacyl synthase domain identified in *G. australes* CAWD149.

| **Seq. Name** | **Seq. Length** | **Blast Hit Description (HSP)** | **Similarity (%)** | **Query Frame** |
| --- | --- | --- | --- | --- |
| 149_contig_46_sl | 3999 | gi\|414091051\|gb\|AFW98413.1\|type I polyketide synthase [*Alexandrium ostenfeldii*] | 53 | 2 |
| 149_contig_243 | 3470 | gi\|414091051\|gb\|AFW98413.1\|type I polyketide synthase [*Alexandrium ostenfeldii*] | 53 | -2 |
| 149_contig_286 | 3522 | gi\|148536473\|gb\|ABQ85796.1\|type I polyketide synthase-like protein KB1008 [*Karenia brevis*] | 52 | -3 |
| 149_contig_351 | 4544 | gi\|414091051\|gb\|AFW98413.1\|type I polyketide synthase [*Alexandrium ostenfeldii*] | 53 | 2 |
| 149_contig_845_polyA | 4033 | gi\|414091051\|gb\|AFW98413.1\|type I polyketide synthase [*Alexandrium ostenfeldii*] | 55 | 1 |
| 149_contig_976 | 2959 | gi\|148536473\|gb\|ABQ85796.1\|type I polyketide synthase-like protein KB1008 [*Karenia brevis*] | 51 | -2 |
| 149_contig_1057 | 3527 | gi\|414091051\|gb\|AFW98413.1\|type I polyketide synthase [*Alexandrium ostenfeldii*] | 51 | -1 |
| 149_contig_1120 | 3702 | gi\|414091051\|gb\|AFW98413.1\|type I polyketide synthase [*Alexandrium ostenfeldii*] | 50 | -1 |
| 149_contig_1424 | 3112 | gi\|414091053\|gb\|AFW98414.1\|type I polyketide synthase [*Heterocapsa triquetra*] | 73 | 3 |
| 149_contig_1645 | 3259 | gi\|148536473\|gb\|ABQ85796.1\|type I polyketide synthase-like protein KB1008 [*Karenia brevis*] | 50 | -1 |
| 149_contig_1672 | 3502 | gi\|148536473\|gb\|ABQ85796.1\|type I polyketide synthase-like protein KB1008 [*Karenia brevis*] | 51 | 1 |
| 149_contig_1728 | 3867 | gi\|414091051\|gb\|AFW98413.1\|type I polyketide synthase [*Alexandrium ostenfeldii*] | 57 | -2 |
| 149_contig_1760_polyA | 3098 | gi\|414091053\|gb\|AFW98414.1\|type I polyketide synthase [*Heterocapsa triquetra*] | 62 | 3 |
| 149_contig_2494_polyA | 3406 | gi\|414091047\|gb\|AFW98411.1\|type I polyketide synthase [*Alexandrium ostenfeldii*] | 58 | 2 |
| 149_contig_2853 | 3533 | gi\|414091049\|gb\|AFW98412.1\|type I polyketide synthase [*Alexandrium ostenfeldii*] | 91 | 1 |
| 149_contig_3128 | 3728 | gi\|148536473\|gb\|ABQ85796.1\|type I polyketide synthase-like protein KB1008 [*Karenia brevis*] | 52 | -2 |
| 149_contig_3191 | 3019 | gi\|148536473\|gb\|ABQ85796.1\|type I polyketide synthase-like protein KB1008 [*Karenia brevis*] | 53 | 1 |
| 149_contig_3662 | 3448 | gi\|148536473\|gb\|ABQ85796.1\|type I polyketide synthase-like protein KB1008 [*Karenia brevis*] | 48 | 1 |
| 149_contig_3719 | 4339 | gi\|148536473\|gb\|ABQ85796.1\|type I polyketide synthase-like protein KB1008 [*Karenia brevis*] | 42 | -3 |
| 149_contig_4085_polyA | 3587 | gi\|148536473\|gb\|ABQ85796.1\|type I polyketide synthase-like protein KB1008 [*Karenia brevis*] | 50 | 3 |
| 149_contig_6121 | 3663 | gi\|148536473\|gb\|ABQ85796.1\|type I polyketide synthase-like protein KB1008 [*Karenia brevis*] | 51 | 2 |
| 149_contig_6342 | 3255 | gi\|414091047\|gb\|AFW98411.1\|type I polyketide synthase [*Alexandrium ostenfeldii*] | 57 | -3 |
| 149_contig_7998 | 3117 | gi\|414091047\|gb\|AFW98411.1\|type I polyketide synthase [*Alexandrium ostenfeldii*] | 84 | -1 |
| 149_contig_8199 | 3145 | gi\|414091049\|gb\|AFW98412.1\|type I polyketide synthase [*Alexandrium ostenfeldii*] | 53 | -2 |
| 149_contig_8241 | 4107 | gi\|414091051\|gb\|AFW98413.1\|type I polyketide synthase [*Alexandrium ostenfeldii*] | 52 | 3 |
| 149_contig_8279 | 4178 | gi\|414091051\|gb\|AFW98413.1\|type I polyketide synthase [*Alexandrium ostenfeldii*] | 54 | 1 |
| 149_contig_8413 | 3604 | gi\|148536473\|gb\|ABQ85796.1\|type I polyketide synthase-like protein KB1008 [*Karenia brevis*] | 46 | 2 |
| 149_contig_8459 | 2995 | gi\|414091053\|gb\|AFW98414.1\|type I polyketide synthase [*Heterocapsa triquetra*] | 62 | -2 |
| 149_contig_9185 | 2987 | gi\|414091047\|gb\|AFW98411.1\|type I polyketide synthase [*Alexandrium ostenfeldii*] | 80 | 2 |
| 149_contig_9390 | 3906 | gi\|148536485\|gb\|ABQ85802.1\|type I polyketide synthase-like protein KB6736 [*Karenia brevis*] | 57 | 3 |
| 149_contig_9558 | 3946 | gi\|414091051\|gb\|AFW98413.1\|type I polyketide synthase [*Alexandrium ostenfeldii*] | 52 | 3 |
| 149_contig_10043 | 3970 | gi\|414091051\|gb\|AFW98413.1\|type I polyketide synthase [*Alexandrium ostenfeldii*] | 57 | 2 |
| 149_contig_10343 | 3845 | gi\|414091051\|gb\|AFW98413.1\|type I polyketide synthase [*Alexandrium ostenfeldii*] | 54 | 3 |
| 149_contig_10413 | 3210 | gi\|148536473\|gb\|ABQ85796.1\|type I polyketide synthase-like protein KB1008 [*Karenia brevis*] | 52 | 2 |
| 149_contig_10435 | 3170 | gi\|414091053\|gb\|AFW98414.1\|type I polyketide synthase [*Heterocapsa triquetra*] | 72 | 2 |
| 149_contig_10820_polyA | 3836 | gi\|414091051\|gb\|AFW98413.1\|type I polyketide synthase [*Alexandrium ostenfeldii*] | 51 | -2 |
| 149_contig_10957_polyA | 3746 | gi\|414091051\|gb\|AFW98413.1\|type I polyketide synthase [*Alexandrium ostenfeldii*] | 58 | 2 |
| 149_contig_11481 | 4057 | gi\|414091051\|gb\|AFW98413.1\|type I polyketide synthase [*Alexandrium ostenfeldii*] | 53 | 2 |
| 149_contig_11807_sl | 3964 | gi\|414091051\|gb\|AFW98413.1\|type I polyketide synthase [*Alexandrium ostenfeldii*] | 50 | 2 |
| 149_contig_11886 | 4018 | gi\|414091051\|gb\|AFW98413.1\|type I polyketide synthase [*Alexandrium ostenfeldii*] | 54 | 3 |
| 149_contig_12750 | 3144 | gi\|148536481\|gb\|ABQ85800.1\|type I polyketide synthase-like protein KB5361 [*Karenia brevis*] | 72 | 1 |
| 149_contig_13107 | 2519 | gi\|414091051\|gb\|AFW98413.1\|type I polyketide synthase [*Alexandrium ostenfeldii*] | 56 | 2 |
| 149_contig_13283 | 3314 | gi\|414091049\|gb\|AFW98412.1\|type I polyketide synthase [*Alexandrium ostenfeldii*] | 59 | 3 |
| 149_contig_13400 | 3555 | gi\|148536485\|gb\|ABQ85802.1\|type I polyketide synthase-like protein KB6736 [*Karenia brevis*] | 51 | 3 |
| 149_contig_14503_polyA | 3108 | gi\|414091049\|gb\|AFW98412.1\|type I polyketide synthase [*Alexandrium ostenfeldii*] | 58 | 3 |
| 149_contig_15813 | 3627 | gi\|148536473\|gb\|ABQ85796.1\|type I polyketide synthase-like protein KB1008 [*Karenia brevis*] | 51 | 3 |
| 149_contig_16171_sl | 3607 | gi\|414091051\|gb\|AFW98413.1\|type I polyketide synthase [*Alexandrium ostenfeldii*] | 52 | -2 |
| 149_contig_16344 | 4165 | gi\|414091051\|gb\|AFW98413.1\|type I polyketide synthase [*Alexandrium ostenfeldii*] | 53 | 1 |
| 149_contig_17011 | 3139 | gi\|414091047\|gb\|AFW98411.1\|type I polyketide synthase [*Alexandrium ostenfeldii*] | 71 | -2 |
| 149_contig_17576_sl | 3368 | gi\|414091051\|gb\|AFW98413.1\|type I polyketide synthase [*Alexandrium ostenfeldii*] | 55 | -1 |
| 149_contig_18730 | 3827 | gi\|414091051\|gb\|AFW98413.1\|type I polyketide synthase [*Alexandrium ostenfeldii*] | 47 | 2 |
| 149_contig_20258 | 3717 | gi\|148536473\|gb\|ABQ85796.1\|type I polyketide synthase-like protein KB1008 [*Karenia brevis*] | 48 | -1 |
| 149_contig_20936 | 3968 | gi\|414091051\|gb\|AFW98413.1\|type I polyketide synthase [*Alexandrium ostenfeldii*] | 54 | -3 |
| 149_contig_21499 | 1742 | gi\|414091049\|gb\|AFW98412.1\|type I polyketide synthase [*Alexandrium ostenfeldii*] | 84 | 1 |
| 149_contig_21616 | 3246 | gi\|148536473\|gb\|ABQ85796.1\|type I polyketide synthase-like protein KB1008 [*Karenia brevis*] | 48 | 3 |
| 149_contig_22541 | 3837 | gi\|414091049\|gb\|AFW98412.1\|type I polyketide synthase [*Alexandrium ostenfeldii*] | 50 | 2 |
| 149_contig_22774 | 3659 | gi\|148536473\|gb\|ABQ85796.1\|type I polyketide synthase-like protein KB1008 [*Karenia brevis*] | 52 | -2 |
| 149_contig_23572 | 3136 | gi\|148536473\|gb\|ABQ85796.1\|type I polyketide synthase-like protein KB1008 [*Karenia brevis*] | 49 | -2 |
| 149_contig_23819 | 3184 | gi\|414091053\|gb\|AFW98414.1\|type I polyketide synthase [*Heterocapsa triquetra*] | 60 | 2 |
| 149_contig_24815_polyA | 3654 | gi\|148536473\|gb\|ABQ85796.1\|type I polyketide synthase-like protein KB1008 [*Karenia brevis*] | 54 | 1 |
| 149_contig_24973_polyA | 2390 | gi\|66359216\|ref\|XP_626786.1\|type I fatty acid synthase [*Cryptosporidium parvum* Iowa II] | 54 | -2 |
| 149_contig_27350 | 3787 | gi\|148536473\|gb\|ABQ85796.1\|type I polyketide synthase-like protein KB1008 [*Karenia brevis*] | 49 | 1 |
| 149_contig_27608 | 3281 | gi\|414091053\|gb\|AFW98414.1\|type I polyketide synthase [*Heterocapsa triquetra*] | 61 | -2 |
| 149_contig_28561 | 2870 | gi\|414091047\|gb\|AFW98411.1\|type I polyketide synthase [*Alexandrium ostenfeldii*] | 75 | 2 |
| 149_contig_29311 | 3101 | gi\|414091053\|gb\|AFW98414.1\|type I polyketide synthase [*Heterocapsa triquetra*] | 54 | 2 |
| 149_contig_30134 | 3911 | gi\|414091051\|gb\|AFW98413.1\|type I polyketide synthase [*Alexandrium ostenfeldii*] | 54 | -3 |
| 149_contig_30642 | 3253 | gi\|148536473\|gb\|ABQ85796.1\|type I polyketide synthase-like protein KB1008 [*Karenia brevis*] | 53 | -3 |
| 149_contig_30846_polyA | 3770 | gi\|148536485\|gb\|ABQ85802.1\|type I polyketide synthase-like protein KB6736 [*Karenia brevis*] | 57 | -2 |
| 149_contig_33479 | 4172 | gi\|148536473\|gb\|ABQ85796.1\|type I polyketide synthase-like protein KB1008 [*Karenia brevis*] | 49 | 3 |
| 149_contig_33766 | 3096 | gi\|148536473\|gb\|ABQ85796.1\|type I polyketide synthase-like protein KB1008 [*Karenia brevis*] | 51 | 1 |
| 149_contig_34708_polyA | 3230 | gi\|148536473\|gb\|ABQ85796.1\|type I polyketide synthase-like protein KB1008 [*Karenia brevis*] | 47 | 2 |
| 149_contig_37305 | 4276 | gi\|148536473\|gb\|ABQ85796.1\|type I polyketide synthase-like protein KB1008 [*Karenia brevis*] | 46 | -3 |
| 149_contig_37306 | 2562 | gi\|148536473\|gb\|ABQ85796.1\|type I polyketide synthase-like protein KB1008 [*Karenia brevis*] | 49 | -2 |
| 149_contig_38837 | 4044 | gi\|148536473\|gb\|ABQ85796.1\|type I polyketide synthase-like protein KB1008 [*Karenia brevis*] | 49 | -3 |
| 149_contig_38849 | 3472 | gi\|414091051\|gb\|AFW98413.1\|type I polyketide synthase [*Alexandrium ostenfeldii*] | 49 | -2 |
| 149_contig_38859 | 3377 | gi\|148536473\|gb\|ABQ85796.1\|type I polyketide synthase-like protein KB1008 [*Karenia brevis*] | 43 | -3 |
| 149_contig_39036_polyA | 2730 | gi\|414091051\|gb\|AFW98413.1\|type I polyketide synthase [*Alexandrium ostenfeldii*] | 88 | -2 |
| 149_contig_39322 | 3985 | gi\|148536485\|gb\|ABQ85802.1\|type I polyketide synthase-like protein KB6736 [*Karenia brevis*] | 55 | 2 |
| 149_contig_41441_polyA | 3894 | gi\|414091051\|gb\|AFW98413.1\|type I polyketide synthase [*Alexandrium ostenfeldii*] | 52 | -1 |
| 149_contig_43205 | 2969 | gi\|414091047\|gb\|AFW98411.1\|type I polyketide synthase [*Alexandrium ostenfeldii*] | 73 | 2 |
| 149_contig_48110 | 3529 | gi\|148536473\|gb\|ABQ85796.1\|type I polyketide synthase-like protein KB1008 [*Karenia brevis*] | 53 | -3 |
| 149_contig_48688 | 2737 | gi\|414091051\|gb\|AFW98413.1\|type I polyketide synthase [*Alexandrium ostenfeldii*] | 52 | 1 |
| 149_contig_49051 | 4066 | gi\|414091051\|gb\|AFW98413.1\|type I polyketide synthase [*Alexandrium ostenfeldii*] | 51 | 1 |
| 149_contig_50669 | 3975 | gi\|148536473\|gb\|ABQ85796.1\|type I polyketide synthase-like protein KB1008 [*Karenia brevis*] | 41 | 1 |
| 149_contig_52057 | 2937 | gi\|414091049\|gb\|AFW98412.1\|type I polyketide synthase [*Alexandrium ostenfeldii*] | 76 | -1 |
| 149_contig_53568 | 3833 | gi\|414091051\|gb\|AFW98413.1\|type I polyketide synthase [*Alexandrium ostenfeldii*] | 51 | -3 |
| 149_contig_57305 | 4456 | gi\|148536485\|gb\|ABQ85802.1\|type I polyketide synthase-like protein KB6736 [*Karenia brevis*] | 49 | 3 |
| 149_contig_58683 | 2691 | gi\|414091051\|gb\|AFW98413.1\|type I polyketide synthase [*Alexandrium ostenfeldii*] | 50 | 2 |

Supplementary table 6: Sequence properties of the transcripts encoding full ketoacyl synthase domain identified in *G. belizeanus.*

| **Seq. Name** | **Seq. Length** | **Blast Hit Description (HSP)** | **Similarity (%)** | **Query Frame** |
| --- | --- | --- | --- | --- |
| 401_contig_231 | 3800 | gi\|414091051\|gb\|AFW98413.1\|type I polyketide synthase [*Alexandrium ostenfeldii*] | 58 | -2 |
| 401_contig_314 | 3865 | gi\|414091051\|gb\|AFW98413.1\|type I polyketide synthase [*Alexandrium ostenfeldii*] | 54 | 2 |
| 401_contig_328_polyA | 3626 | gi\|148536473\|gb\|ABQ85796.1\|type I polyketide synthase-like protein KB1008 [*Karenia brevis*] | 53 | -1 |
| 401_contig_740 | 2649 | gi\|414091051\|gb\|AFW98413.1\|type I polyketide synthase [*Alexandrium ostenfeldii*] | 88 | 1 |
| 401_contig_1592 | 3433 | gi\|148536473\|gb\|ABQ85796.1\|type I polyketide synthase-like protein KB1008 [*Karenia brevis*] | 48 | -1 |
| 401_contig_1754_polyA | 3357 | gi\|148536473\|gb\|ABQ85796.1\|type I polyketide synthase-like protein KB1008 [*Karenia brevis*] | 53 | 2 |
| 401_contig_3368 | 3124 | gi\|414091053\|gb\|AFW98414.1\|type I polyketide synthase [*Heterocapsa triquetra*] | 73 | -3 |
| 401_contig_3740 | 2889 | gi\|414091047\|gb\|AFW98411.1\|type I polyketide synthase [*Alexandrium ostenfeldii*] | 73 | 3 |
| 401_contig_4641 | 4326 | gi\|414091051\|gb\|AFW98413.1\|type I polyketide synthase [*Alexandrium ostenfeldii*] | 54 | 2 |
| 401_contig_4657 | 3262 | gi\|414091049\|gb\|AFW98412.1\|type I polyketide synthase [*Alexandrium ostenfeldii*] | 48 | -2 |
| 401_contig_4850 | 3151 | gi\|414091049\|gb\|AFW98412.1\|type I polyketide synthase [*Alexandrium ostenfeldii*] | 92 | 3 |
| 401_contig_5033 | 3016 | gi\|414091047\|gb\|AFW98411.1\|type I polyketide synthase [*Alexandrium ostenfeldii*] | 71 | -2 |
| 401_contig_5716 | 3645 | gi\|148536473\|gb\|ABQ85796.1\|type I polyketide synthase-like protein KB1008 [*Karenia brevis*] | 51 | 3 |
| 401_contig_6727_polyA | 3669 | gi\|148536473\|gb\|ABQ85796.1\|type I polyketide synthase-like protein KB1008 [*Karenia brevis*] | 52 | -3 |
| 401_contig_7263 | 3985 | gi\|414091051\|gb\|AFW98413.1\|type I polyketide synthase [*Alexandrium ostenfeldii*] | 53 | 1 |
| 401_contig_7842 | 3211 | gi\|414091047\|gb\|AFW98411.1\|type I polyketide synthase [*Alexandrium ostenfeldii*] | 58 | -2 |
| 401_contig_9104 | 3605 | gi\|148536473\|gb\|ABQ85796.1\|type I polyketide synthase-like protein KB1008 [*Karenia brevis*] | 51 | -2 |
| 401_contig_9638 | 2855 | gi\|414091051\|gb\|AFW98413.1\|type I polyketide synthase [*Alexandrium ostenfeldii*] | 53 | 3 |
| 401_contig_10081_polyA | 3253 | gi\|414091053\|gb\|AFW98414.1\|type I polyketide synthase [*Heterocapsa triquetra*] | 59 | 1 |
| 401_contig_10472 | 2996 | gi\|148536473\|gb\|ABQ85796.1\|type I polyketide synthase-like protein KB1008 [*Karenia brevis*] | 54 | 3 |
| 401_contig_10518 | 2950 | gi\|414091047\|gb\|AFW98411.1\|type I polyketide synthase [*Alexandrium ostenfeldii*] | 81 | 1 |
| 401_contig_11261_polyA | 3173 | gi\|148536473\|gb\|ABQ85796.1\|type I polyketide synthase-like protein KB1008 [*Karenia brevis*] | 49 | -1 |
| 401_contig_11434 | 4241 | gi\|414091051\|gb\|AFW98413.1\|type I polyketide synthase [*Alexandrium ostenfeldii*] | 56 | -1 |
| 401_contig_12018 | 3180 | gi\|148536473\|gb\|ABQ85796.1\|type I polyketide synthase-like protein KB1008 [*Karenia brevis*] | 47 | 3 |
| 401_contig_13526_polyA | 2727 | gi\|67615114\|ref\|XP_667412.1\|hypothetical protein [*Cryptosporidium hominis* TU502] | 54 | 1 |
| 401_contig_14065 | 3979 | gi\|414091051\|gb\|AFW98413.1\|type I polyketide synthase [*Alexandrium ostenfeldii*] | 53 | 3 |
| 401_contig_14246 | 4010 | gi\|414091051\|gb\|AFW98413.1\|type I polyketide synthase [*Alexandrium ostenfeldii*] | 52 | -1 |
| 401_contig_14264 | 2474 | gi\|414091049\|gb\|AFW98412.1\|type I polyketide synthase [*Alexandrium ostenfeldii*] | 56 | 2 |
| 401_contig_15627 | 4005 | gi\|148536485\|gb\|ABQ85802.1\|type I polyketide synthase-like protein KB6736 [*Karenia brevis*] | 56 | 3 |
| 401_contig_16114 | 3919 | gi\|414091051\|gb\|AFW98413.1\|type I polyketide synthase [*Alexandrium ostenfeldii*] | 52 | 2 |
| 401_contig_16126_polyA | 3140 | gi\|414091049\|gb\|AFW98412.1\|type I polyketide synthase [*Alexandrium ostenfeldii*] | 59 | 1 |
| 401_contig_16876_polyA | 2938 | gi\|148536473\|gb\|ABQ85796.1\|type I polyketide synthase-like protein KB1008 [*Karenia brevis*] | 50 | 1 |
| 401_contig_17392__SL-polyA | 3896 | gi\|414091051\|gb\|AFW98413.1\|type I polyketide synthase [*Alexandrium ostenfeldii*] | 47 | 2 |
| 401_contig_18839_polyA | 3137 | gi\|148536473\|gb\|ABQ85796.1\|type I polyketide synthase-like protein KB1008 [*Karenia brevis*] | 43 | 3 |
| 401_contig_18880 | 4114 | gi\|414091051\|gb\|AFW98413.1\|type I polyketide synthase [*Alexandrium ostenfeldii*] | 54 | 3 |
| 401_contig_19545 | 4046 | gi\|148536473\|gb\|ABQ85796.1\|type I polyketide synthase-like protein KB1008 [*Karenia brevis*] | 48 | -3 |
| 401_contig_21075 | 4078 | gi\|414091051\|gb\|AFW98413.1\|type I polyketide synthase [*Alexandrium ostenfeldii*] | 52 | -2 |
| 401_contig_21212 | 2690 | gi\|148536473\|gb\|ABQ85796.1\|type I polyketide synthase-like protein KB1008 [*Karenia brevis*] | 50 | 2 |
| 401_contig_21746 | 3598 | gi\|148536473\|gb\|ABQ85796.1\|type I polyketide synthase-like protein KB1008 [*Karenia brevis*] | 48 | -2 |
| 401_contig_22426 | 3139 | gi\|414091053\|gb\|AFW98414.1\|type I polyketide synthase [*Heterocapsa triquetra*] | 63 | 1 |
| 401_contig_23204 | 4046 | gi\|414091051\|gb\|AFW98413.1\|type I polyketide synthase [*Alexandrium ostenfeldii*] | 54 | 2 |
| 401_contig_23479 | 3649 | gi\|148536473\|gb\|ABQ85796.1\|type I polyketide synthase-like protein KB1008 [*Karenia brevis*] | 50 | 3 |
| 401_contig_24093 | 3476 | gi\|414091053\|gb\|AFW98414.1\|type I polyketide synthase [*Heterocapsa triquetra*] | 62 | 2 |
| 401_contig_24439_polyA | 4043 | gi\|414091051\|gb\|AFW98413.1\|type I polyketide synthase [*Alexandrium ostenfeldii*] | 53 | 1 |
| 401_contig_24708 | 3820 | gi\|414091051\|gb\|AFW98413.1\|type I polyketide synthase [*Alexandrium ostenfeldii*] | 55 | -1 |
| 401_contig_25000 | 3825 | gi\|414091051\|gb\|AFW98413.1\|type I polyketide synthase [*Alexandrium ostenfeldii*] | 57 | 1 |
| 401_contig_25684 | 3038 | gi\|414091053\|gb\|AFW98414.1\|type I polyketide synthase [*Heterocapsa triquetra*] | 62 | -2 |
| 401_contig_27576 | 3112 | gi\|414091049\|gb\|AFW98412.1\|type I polyketide synthase [*Alexandrium ostenfeldii*] | 59 | -2 |
| 401_contig_28155_polyA | 3053 | gi\|414091047\|gb\|AFW98411.1\|type I polyketide synthase [*Alexandrium ostenfeldii*] | 85 | -1 |
| 401_contig_28804 | 3619 | gi\|414091051\|gb\|AFW98413.1\|type I polyketide synthase [*Alexandrium ostenfeldii*] | 50 | -3 |
| 401_contig_28978 | 2788 | gi\|414091051\|gb\|AFW98413.1\|type I polyketide synthase [*Alexandrium ostenfeldii*] | 49 | 1 |
| 401_contig_29863 | 3846 | gi\|148536485\|gb\|ABQ85802.1\|type I polyketide synthase-like protein KB6736 [*Karenia brevis*] | 56 | -2 |
| 401_contig_29972 | 2576 | gi\|414091051\|gb\|AFW98413.1\|type I polyketide synthase [*Alexandrium ostenfeldii*] | 53 | 1 |
| 401_contig_31749 | 1620 | gi\|414091047\|gb\|AFW98411.1\|type I polyketide synthase [*Alexandrium ostenfeldii*] | 60 | -3 |
| 401_contig_36046 | 3112 | gi\|148536481\|gb\|ABQ85800.1\|type I polyketide synthase-like protein KB5361 [*Karenia brevis*] | 72 | 2 |
| 401_contig_36166 | 2961 | gi\|414091053\|gb\|AFW98414.1\|type I polyketide synthase [*Heterocapsa triquetra*] | 62 | -2 |
| 401_contig_36733 | 3709 | gi\|148536473\|gb\|ABQ85796.1\|type I polyketide synthase-like protein KB1008 [*Karenia brevis*] | 54 | 1 |
| 401_contig_37136_polyA | 3196 | gi\|148536473\|gb\|ABQ85796.1\|type I polyketide synthase-like protein KB1008 [*Karenia brevis*] | 50 | 2 |
| 401_contig_38628_polyA | 3918 | gi\|414091051\|gb\|AFW98413.1\|type I polyketide synthase [*Alexandrium ostenfeldii*] | 52 | 2 |
| 401_contig_39242 | 2812 | gi\|148536485\|gb\|ABQ85802.1\|type I polyketide synthase-like protein KB6736 [*Karenia brevis*] | 45 | -2 |
| 401_contig_42631 | 3955 | gi\|414091051\|gb\|AFW98413.1\|type I polyketide synthase [*Alexandrium ostenfeldii*] | 52 | 1 |
| 401_contig_43235 | 3039 | gi\|414091053\|gb\|AFW98414.1\|type I polyketide synthase [*Heterocapsa triquetra*] | 53 | 1 |
| 401_contig_44574 | 3781 | gi\|148536485\|gb\|ABQ85802.1\|type I polyketide synthase-like protein KB6736 [*Karenia brevis*] | 58 | 1 |
| 401_contig_45047 | 3353 | gi\|148536473\|gb\|ABQ85796.1\|type I polyketide synthase-like protein KB1008 [*Karenia brevis*] | 41 | 3 |
| 401_contig_47689 | 3820 | gi\|148536473\|gb\|ABQ85796.1\|type I polyketide synthase-like protein KB1008 [*Karenia brevis*] | 48 | -2 |
| 401_contig_48690 | 2765 | gi\|414091051\|gb\|AFW98413.1\|type I polyketide synthase [*Alexandrium ostenfeldii*] | 54 | 1 |
| 401_contig_49748 | 3623 | gi\|414091051\|gb\|AFW98413.1\|type I polyketide synthase [*Alexandrium ostenfeldii*] | 57 | -3 |
| 401_contig_50159_polyA | 4257 | gi\|414091051\|gb\|AFW98413.1\|type I polyketide synthase [*Alexandrium ostenfeldii*] | 48 | 2 |
| 401_contig_56915_polyA | 3771 | gi\|414091051\|gb\|AFW98413.1\|type I polyketide synthase [*Alexandrium ostenfeldii*] | 56 | -2 |
| 401_contig_59044 | 3184 | gi\|148536473\|gb\|ABQ85796.1\|type I polyketide synthase-like protein KB1008 [*Karenia brevis*] | 54 | 2 |
| 401_contig_60106 | 1763 | gi\|148536473\|gb\|ABQ85796.1\|type I polyketide synthase-like protein KB1008 [*Karenia brevis*] | 49 | -3 |
| 401_contig_62184 | 3622 | gi\|148536473\|gb\|ABQ85796.1\|type I polyketide synthase-like protein KB1008 [*Karenia brevis*] | 50 | -3 |
| 401_contig_76411 | 1910 | gi\|414091049\|gb\|AFW98412.1\|type I polyketide synthase [*Alexandrium ostenfeldii*] | 70 | 3 |
| 401_contig_79075 | 1923 | gi\|414091047\|gb\|AFW98411.1\|type I polyketide synthase [*Alexandrium ostenfeldii*] | 79 | 3 |

Supplementary table 7: Sequence properties of the transcripts encoding partial ketoacyl synthase domain identified in *G. australes* (sequence name starting with 149) and *G. belizeanus* (sequence name starting with 401).

| **Seq. Name** | **Seq. Length** | **Blast Hit Description (HSP)** | **Similarity (%)** | **Query Frame** |
| --- | --- | --- | --- | --- |
| 149_contig_2649 | 2861 | gi\|148536473\|gb\|ABQ85796.1\|type I polyketide synthase-like protein KB1008 [*Karenia brevis*] | 44 | -2 |
| 149_contig_7195 | 1998 | gi\|414091053\|gb\|AFW98414.1\|type I polyketide synthase [*Heterocapsa triquetra*] | 64 | -2 |
| 149_contig_22400 | 1945 | gi\|148536479\|gb\|ABQ85799.1\|type I polyketide synthase-like protein KB5299 [*Karenia brevis*] | 42 | 3 |
| 149_contig_31995 | 4320 | gi\|414091051\|gb\|AFW98413.1\|type I polyketide synthase [*Alexandrium ostenfeldii*] | 47 | -2 |
| 149_contig_40561 | 1283 | gi\|148536473\|gb\|ABQ85796.1\|type I polyketide synthase-like protein KB1008 [*Karenia brevis*] | 50 | -2 |
| 149_contig_47966 | 4199 | gi\|414091051\|gb\|AFW98413.1\|type I polyketide synthase [*Alexandrium ostenfeldii*] | 48 | 2 |
| 401_contig_4413 | 2127 | gi\|414091051\|gb\|AFW98413.1\|type I polyketide synthase [*Alexandrium ostenfeldii*] | 52 | -3 |
| 401_contig_7439_polyA | 1629 | gi\|148536483\|gb\|ABQ85801.1\|type I polyketide synthase-like protein KB6380 [*Karenia brevis*] | 47 | 1 |
| 401_contig_8665_polyA | 1587 | gi\|148536483\|gb\|ABQ85801.1\|type I polyketide synthase-like protein KB6380 [*Karenia brevis*] | 44 | 1 |
| 401_contig_15573 | 2381 | gi\|148536485\|gb\|ABQ85802.1\|type I polyketide synthase-like protein KB6736 [*Karenia brevis*] | 52 | -1 |
| 401_contig_23320 | 1825 | gi\|148536473\|gb\|ABQ85796.1\|type I polyketide synthase-like protein KB1008 [*Karenia brevis*] | 44 | 1 |
| 401_contig_25337 | 1730 | gi\|414091053\|gb\|AFW98414.1\|type I polyketide synthase [*Heterocapsa triquetra*] | 77 | -3 |
| 401_contig_26706 | 2471 | gi\|148536473\|gb\|ABQ85796.1\|type I polyketide synthase-like protein KB1008 [*Karenia brevis*] | 44 | 1 |
| 401_contig_30882 | 1083 | gi\|414091051\|gb\|AFW98413.1\|type I polyketide synthase [*Alexandrium ostenfeldii*] | 51 | -2 |
| 401_contig_36150_polyA | 1956 | gi\|148536485\|gb\|ABQ85802.1\|type I polyketide synthase-like protein KB6736 [*Karenia brevis*] | 52 | 2 |
| 401_contig_43131 | 970 | gi\|148536487\|gb\|ABQ85803.1\|type I polyketide synthase-like protein KB6842 [*Karenia brevis*] | 51 | -2 |
| 401_contig_43437 | 1908 | gi\|414091051\|gb\|AFW98413.1\|type I polyketide synthase [*Alexandrium ostenfeldii*] | 47 | 2 |
| 401_contig_48644 | 1760 | gi\|148536473\|gb\|ABQ85796.1\|type I polyketide synthase-like protein KB1008 [*Karenia brevis*] | 41 | -3 |
| 401_contig_62265 | 878 | gi\|401395940\|ref\|XP_003879716.1\|short-chain dehydrogenase/reductase SDR, related [*Neospora caninum* Liverpool] | 55 | 2 |
| 401_contig_64415 | 1939 | gi\|148536485\|gb\|ABQ85802.1\|type I polyketide synthase-like protein KB6736 [*Karenia brevis*] | 46 | 3 |
| 401_contig_64549 | 1652 | gi\|414091051\|gb\|AFW98413.1\|type I polyketide synthase [*Alexandrium ostenfeldii*] | 47 | 2 |
| 401_contig_68206 | 904 | gi\|148536473\|gb\|ABQ85796.1\|type I polyketide synthase-like protein KB1008 [*Karenia brevis*] | 56 | -2 |
| 401_contig_69866 | 1660 | gi\|414091049\|gb\|AFW98412.1\|type I polyketide synthase [*Alexandrium ostenfeldii*] | 82 | 1 |
| 401_contig_73178 | 2000 | gi\|148536473\|gb\|ABQ85796.1\|type I polyketide synthase-like protein KB1008 [*Karenia brevis*] | 42 | -3 |
| 401_contig_78386 | 1268 | gi\|148536475\|gb\|ABQ85797.1\|type I polyketide synthase-like protein KB2006 [*Karenia brevis*] | 43 | -1 |
| 401_contig_79490 | 479 | gi\|414091049\|gb\|AFW98412.1\|type I polyketide synthase [*Alexandrium ostenfeldii*] | 77 | 1 |
| 401_contig_82561_polyA | 1038 | gi\|148536473\|gb\|ABQ85796.1\|type I polyketide synthase-like protein KB1008 [*Karenia brevis*] | 53 | -3 |
| 401_contig_82587 | 872 | gi\|148536473\|gb\|ABQ85796.1\|type I polyketide synthase-like protein KB1008 [*Karenia brevis*] | 50 | 2 |
| 401_contig_91363 | 1420 | gi\|414091051\|gb\|AFW98413.1\|type I polyketide synthase [*Alexandrium ostenfeldii*] | 47 | 3 |
| 401_contig_104824 | 454 | gi\|209880347\|ref\|XP_002141613.1\|type I fatty acid synthase [*Cryptosporidium muris* RN66] | 60 | 2 |

Supplementary table 8: Sequence properties of the transcripts encoding full and partial ketoyl reductase domains identified in *G. australes* (sequence name starting with 149) and *G. belizeanus* (sequence name starting with 401).

| **Seq. Name** | **Seq. Length** | **Blast Hit Description (HSP)** | **Similarity (%)** | **Query Frame** |
| --- | --- | --- | --- | --- |
| 149_contig_1012 | 1952 | gi\|193891021\|gb\|ACF28664.1\|ketoyl reductase domain protein [*Amphidinium carterae*] | 54 | 1 |
| 149_contig_4556 | 2151 | gi\|193891021\|gb\|ACF28664.1\|ketoyl reductase domain protein [*Amphidinium carterae*] | 55 | -1 |
| 149_contig_7027_polyA | 2313 | gi\|193891021\|gb\|ACF28664.1\|ketoyl reductase domain protein [*Amphidinium carterae*] | 55 | 3 |
| 149_contig_11318_sl | 3758 | gi\|193891021\|gb\|ACF28664.1\|ketoyl reductase domain protein [*Amphidinium carterae*] | 52 | 1 |
| 149_contig_16944_sl | 1155 | gi\|485632707\|gb\|EOD27672.1\| chloroplast beta-keto acyl reductase [*Emiliania huxleyi* CCMP1516] | 75 | -1 |
| 149_contig_23063_sl | 2120 | gi\|193891021\|gb\|ACF28664.1\|ketoyl reductase domain protein [*Amphidinium carterae*] | 54 | 2 |
| 149_contig_34033 | 2181 | gi\|193891021\|gb\|ACF28664.1\|ketoyl reductase domain protein [*Amphidinium carterae*] | 51 | -2 |
| 149_contig_61256 | 1246 | gi\|193891021\|gb\|ACF28664.1\|ketoyl reductase domain protein [*Amphidinium carterae*] | 43 | 3 |
| 401_contig_12357 | 2007 | gi\|193891021\|gb\|ACF28664.1\|ketoyl reductase domain protein [*Amphidinium carterae*] | 51 | -3 |
| 401_contig_20827 | 3682 | gi\|193891021\|gb\|ACF28664.1\|ketoyl reductase domain protein [*Amphidinium carterae*] | 54 | 2 |
| 401_contig_24494_polyA | 2105 | gi\|193891021\|gb\|ACF28664.1\|ketoyl reductase domain protein [*Amphidinium carterae*] | 53 | -1 |
| 401_contig_37710_polyA | 1978 | gi\|193891021\|gb\|ACF28664.1\|ketoyl reductase domain protein [*Amphidinium carterae*] | 54 | 1 |
| 401_contig_33495_polyA | 2009 | gi\|193891021\|gb\|ACF28664.1\|ketoyl reductase domain protein [*Amphidinium carterae*] | 54 | -3 |
| 401_contig_79875 | 1968 | gi\|193891021\|gb\|ACF28664.1\|ketoyl reductase domain protein [*Amphidinium carterae*] | 55 | -3 |
| 149_contig_27636 | 547 | gi\|193890993\|gb\|ACF28650.1\|ketoyl reductase domain protein [*Amphidinium carterae*] | 67 | 1 |
| 149_contig_15234 | 2004 | gi\|407919251\|gb\|EKG12504.1\|Beta-ketoacyl synthase, partial [Macrophomina phaseolina MS6] | 52 | -3 |
| 401_contig_3571 | 2161 | gi\|485647436\|gb\|EOD40598.1\|polyketide synthase [*Emiliania huxleyi* CCMP1516] | 47 | 1 |

Supplementary table 9: Sequence properties of the transcripts encoding full acyl carrier protein synthase, enoylreductase, acyltransferase, acyltransferase with ankyrin adaptor proteins, acyl carrier protein domains identified in *G. australes* (seq. name starting with 149) and *G. belizeanus* (seq. name starting with 401).

| **Seq. Name** | **Seq. Length** | **Blast Hit Description (HSP)** | **Similarity (%)** | **Query Frame** |
| --- | --- | --- | --- | --- |
| **Acyl carrier protein synthase** | | | | |
| 149_contig_1180 | 1854 | gi\|224013261\|ref\|XP_002295282.1\|predicted protein [*Thalassiosira pseudonana* CCMP1335] | 83 | -2 |
| 149_contig_43281 | 4112 | gi\|299473416\|emb\|CBN77814.1\|beta-ketoacyl synthase [*Ectocarpus siliculosus*] | 68 | 3 |
| 401_contig_9135 | 1834 | gi\|224013261\|ref\|XP_002295282.1\|predicted protein [*Thalassiosira pseudonana* CCMP1335] | 83 | -2 |
| **Methyltransferases** | | | | |
| 149_contig_35933 | 1207 | gi\|294901177\|ref\|XP_002777273.1\|3-demethylubiquinone-9 3-methyltransferase, putative [*Perkinsus marinus* ATCC 50983] | 63 | -2 |
| 401_contig_23201_polyA | 1253 | gi\|294901177\|ref\|XP_002777273.1\|3-demethylubiquinone-9 3-methyltransferase, putative [*Perkinsus marinus* ATCC 50983] | 61 | 2 |
| 149_contig_71891_polyA | 1213 | gi\|412992361\|emb\|CCO20074.1\|Thioesterase [*Bathycoccus prasinos*] | 45 | 1 |
| 149_contig_6907 | 3740 | gi\|460409447\|ref\|XP_004250150.1\|PREDICTED: protein SGT1 homolog At5g65490-like [*Solanum lycopersicum*] | 44 | -1 |
| 401_contig_14318 | 1378 | gi\|412992361\|emb\|CCO20074.1\|Thioesterase [*Bathycoccus prasinos*] | 46 | 2 |
| 401_contig_18809 | 1327 | gi\|308799841\|ref\|XP_003074702.1\|COG3208: thioesterase involved in non-ri (ISS) [*Ostreococcus tauri*] | 50 | -2 |
| 401_contig_59297_polyA | 1185 | gi\|412992361\|emb\|CCO20074.1\|Thioesterase [*Bathycoccus prasinos*] | 45 | 2 |
| **Enoylreductases** | | | | |
| 401_contig_95883 | 1065 | gi\|237841907\|ref\|XP_002370251.1\|type I fatty acid synthase, putative [*Toxoplasma gondii* ME49] | 66 | -2 |
| **Acyltransferases** | | | | |
| 149_contig_1915 | 1846 | gi\|255084229\|ref\|XP_002508689.1\|predicted protein [*Micromonas* sp. RCC299] | 63 | -3 |
| 149_contig_6178_polyA | 1547 | gi\|255084229\|ref\|XP_002508689.1\|predicted protein [*Micromonas* sp. RCC299] | 64 | 3 |
| 149_contig_8353 | 1854 | gi\|307108213\|gb\|EFN56454.1\|hypothetical protein CHLNCDRAFT_22513 [*Chlorella variabilis*] | 62 | -1 |
| 149_contig_10903 | 1268 | gi\|307108213\|gb\|EFN56454.1\|hypothetical protein CHLNCDRAFT_22513 [*Chlorella variabilis*] | 63 | -2 |
| 149_contig_11913_polyA | 1700 | gi\|307108213\|gb\|EFN56454.1\|hypothetical protein CHLNCDRAFT_22513 [*Chlorella variabilis*] | 62 | 3 |
| 149_contig_19065_polyA | 1807 | gi\|307108213\|gb\|EFN56454.1\|hypothetical protein CHLNCDRAFT_22513 [*Chlorella variabilis*] | 66 | 2 |
| 149_contig_24465 | 1320 | gi\|529274250\|gb\|AGS78408.1\|malonyl-CoA:Acyl carrier protein transacylase [*Nannochloropsis gaditana*] | 81 | -1 |
| 149_contig_26032 | 3085 | gi\|255084229\|ref\|XP_002508689.1\|predicted protein [*Micromonas* sp. RCC299] | 66 | 3 |
| 149_contig_44455 | 1433 | gi\|255084229\|ref\|XP_002508689.1\|predicted protein [*Micromonas* sp. RCC299] | 59 | -1 |
| 149_contig_45454 | 1551 | gi\|545363729\|ref\|XP_005646973.1\|malonyl-CoA:ACP transacylase [*Coccomyxa subellipsoidea* C-169] | 53 | -1 |
| 149_contig_45580 | 2054 | gi\|255069969\|ref\|XP_002507066.1\|malonyl-CoA:ACP transacylase [*Micromonas* sp. RCC299] | 50 | 2 |
| 149_contig_45715 | 1768 | gi\|545363729\|ref\|XP_005646973.1\|malonyl-CoA:ACP transacylase [*Coccomyxa subellipsoidea* C-169 | 57 | 3 |
| 149_contig_47728 | 1429 | gi\|333036651\|gb\|AEF13163.1\|malonyl coenzyme A acyl carrier protein transacylase [*Haematococcus pluvialis*] | 53 | 3 |
| 149_contig_22951 | 1148 | gi\|255084229\|ref\|XP_002508689.1\|predicted protein [*Micromonas* sp. RCC299] | 56 | 2 |
| 149_contig_6157 | 1831 | gi\|255084229\|ref\|XP_002508689.1\|predicted protein [*Micromonas* sp. RCC299] | 62 | -3 |
| 149_contig_50788 | 766 | gi\|307108213\|gb\|EFN56454.1\|hypothetical protein CHLNCDRAFT_22513 [*Chlorella variabilis*] | 60 | 1 |
| 149_contig_22327 | 746 | gi\|255084229\|ref\|XP_002508689.1\|predicted protein [*Micromonas* sp. RCC299] | 69 | -1 |
| 401_contig_941 | 1892 | gi\|307108213\|gb\|EFN56454.1\|hypothetical protein CHLNCDRAFT_22513 [*Chlorella variabilis*] | 62 | 2 |
| 401_contig_2993 | 1804 | gi\|545363729\|ref\|XP_005646973.1\|malonyl-CoA:ACP transacylase [*Coccomyxa subellipsoidea* C-169] | 62 | 3 |
| 401_contig_4897_polyA | 1117 | gi\|255084229\|ref\|XP_002508689.1\|predicted protein [*Micromonas* sp. RCC299] | 63 | -3 |
| 401_contig_7825 | 1214 | gi\|547913332\|ref\|WP_022316201.1\|malonyl CoA-acyl carrier protein transacylase [*Prevotella sp.* CAG:592] | 51 | 1 |
| 401_contig_9245 | 1520 | gi\|255084229\|ref\|XP_002508689.1\|predicted protein [*Micromonas* sp. RCC299] | 60 | -2 |
| 401_contig_18262 | 1121 | gi\|545363729\|ref\|XP_005646973.1\|malonyl-CoA:ACP transacylase [*Coccomyxa subellipsoidea* C-169] | 63 | -1 |
| 401_contig_22128 | 946 | gi\|545363729\|ref\|XP_005646973.1\|malonyl-CoA:ACP transacylase [*Coccomyxa subellipsoidea* C-169] | 63 | 3 |
| 401_contig_23849 | 1576 | gi\|255084229\|ref\|XP_002508689.1\|predicted protein [*Micromonas* sp. RCC299] | 60 | -3 |
| 401_contig_27668_polyA | 1156 | gi\|307108213\|gb\|EFN56454.1\|hypothetical protein CHLNCDRAFT_22513 [*Chlorella variabilis*] | 63 | -3 |
| 401_contig_28503 | 1061 | gi\|307108213\|gb\|EFN56454.1\|hypothetical protein CHLNCDRAFT_22513 [*Chlorella variabilis*] | 61 | -3 |
| 401_contig_28751 | 2114 | gi\|255069969\|ref\|XP_002507066.1\|malonyl-CoA:ACP transacylase [*Micromonas* sp. RCC299] | 53 | 1 |
| 401_contig_29704 | 1197 | gi\|255084229\|ref\|XP_002508689.1\|predicted protein [*Micromonas* sp. RCC299] | 63 | -2 |
| 401_contig_37285 | 1263 | gi\|529274250\|gb\|AGS78408.1\|malonyl-CoA:Acyl carrier protein transacylase [*Nannochloropsis gaditana*] | 81 | -3 |
| 401_contig_38101_polyA | 3162 | gi\|255084229\|ref\|XP_002508689.1\|predicted protein [*Micromonas* sp. RCC299] | 64 | 2 |
| 401_contig_38888_polyA | 1684 | gi\|545363729\|ref\|XP_005646973.1\|malonyl-CoA:ACP transacylase [*Coccomyxa subellipsoidea* C-169] | 65 | -2 |
| 401_contig_43348 | 1268 | gi\|255084229\|ref\|XP_002508689.1\|predicted protein [*Micromonas* sp. RCC299] | 50 | -3 |
| 401_contig_52083 | 1249 | gi\|255084229\|ref\|XP_002508689.1\|predicted protein [*Micromonas* sp. RCC299] | 54 | 2 |
| 401_contig_59597 | 849 | gi\|307108213\|gb\|EFN56454.1\|hypothetical protein CHLNCDRAFT_22513 [*Chlorella variabilis*] | 61 | -2 |
| 401_contig_64954 | 1002 | gi\|545363729\|ref\|XP_005646973.1\|malonyl-CoA:ACP transacylase [*Coccomyxa subellipsoidea* C-169] | 58 | 2 |
| 401_contig_72828 | 2696 | gi\|485644237\|gb\|EOD38195.1\|malonyl-CoA:ACP transacylase [*Emiliania huxleyi* CCMP1516] | 51 | 1 |
| 401_contig_83536 | 778 | gi\|307108213\|gb\|EFN56454.1\|hypothetical protein CHLNCDRAFT_22513 [*Chlorella variabilis*] | 59 | 2 |
| **Acyltransferase with ankyrin adaptor proteins** | | | | |
| 149_contig_5204 | 1954 | gi\|545363729\|ref\|XP_005646973.1\|malonyl-CoA:ACP transacylase [*Coccomyxa subellipsoidea* C-169] | 62 | -2 |
| 149_contig_17583 | 1782 | gi\|545363729\|ref\|XP_005646973.1\|malonyl-CoA:ACP transacylase [*Coccomyxa subellipsoidea* C-169] | 65 | 1 |
| 149_contig_37408 | 1037 | gi\|545363729\|ref\|XP_005646973.1\|malonyl-CoA:ACP transacylase [*Coccomyxa subellipsoidea* C-169] | 66 | -1 |
| 149_contig_25153 | 1883 | gi\|545363729\|ref\|XP_005646973.1\|malonyl-CoA:ACP transacylase [*Coccomyxa subellipsoidea* C-169] | 51 | 3 |
| 149_contig_4169 | 1747 | gi\|545363729\|ref\|XP_005646973.1\|malonyl-CoA:ACP transacylase [*Coccomyxa subellipsoidea* C-169] | 62 | 1 |
| 149_contig_26352 | 2007 | gi\|545363729\|ref\|XP_005646973.1\|malonyl-CoA:ACP transacylase [*Coccomyxa subellipsoidea* C-169] | 60 | 1 |
| 149_contig_789_polyAl | 1730 | gi\|545363729\|ref\|XP_005646973.1\|malonyl-CoA:ACP transacylase [*Coccomyxa subellipsoidea* C-169] | 62 | 1 |
| 149_contig_4161 | 1606 | gi\|545363729\|ref\|XP_005646973.1\|malonyl-CoA:ACP transacylase [*Coccomyxa subellipsoidea* C-169] | 60 | 3 |
| 149_contig_36302 | 1609 | gi\|545363729\|ref\|XP_005646973.1\|malonyl-CoA:ACP transacylase [*Coccomyxa subellipsoidea* C-169] | 61 | 1 |
| 149_contig_13421 | 1641 | gi\|255084229\|ref\|XP_002508689.1\|predicted protein [*Micromonas* sp. RCC299] | 59 | 2 |
| 149_contig_53554 | 1133 | gi\|255084229\|ref\|XP_002508689.1\|predicted protein [*Micromonas* sp. RCC299] | 67 | 2 |
| 149_contig_291_sl | 2026 | gi\|255084229\|ref\|XP_002508689.1\|predicted protein [*Micromonas* sp. RCC299] | 56 | -1 |
| 149_contig_7046 | 1764 | gi\|255084229\|ref\|XP_002508689.1\|predicted protein [*Micromonas* sp. RCC299] | 62 | -3 |
| 401_contig_2085 | 1895 | gi\|545363729\|ref\|XP_005646973.1\|malonyl-CoA:ACP transacylase [*Coccomyxa subellipsoidea* C-169] | 50 | -1 |
| 401_contig_8567 | 1139 | gi\|255084229\|ref\|XP_002508689.1\|predicted protein [*Micromonas* sp. RCC299] | 71 | -3 |
| 401_contig_9654 | 1794 | gi\|255084229\|ref\|XP_002508689.1\|predicted protein [*Micromonas* sp. RCC299] | 64 | 2 |
| 401_contig_12997 | 1881 | gi\|307108213\|gb\|EFN56454.1\|hypothetical protein CHLNCDRAFT_22513 [*Chlorella variabilis*] | 60 | 2 |
| 401_contig_13961 | 1500 | gi\|303287712\|ref\|XP_003063145.1\|predicted protein [*Micromonas pusilla* CCMP1545] | 60 | -2 |
| 401_contig_15254 | 1708 | gi\|255084229\|ref\|XP_002508689.1\|predicted protein [*Micromonas* sp. RCC299] | 61 | 3 |
| 401_contig_24372 | 1802 | gi\|307108213\|gb\|EFN56454.1\|hypothetical protein CHLNCDRAFT_22513 [*Chlorella variabilis*] | 61 | 2 |
| 401_contig_31279 | 1303 | gi\|545363729\|ref\|XP_005646973.1\|malonyl-CoA:ACP transacylase [*Coccomyxa subellipsoidea* C-169] | 65 | -3 |
| **Acyl carrier protein** | | | | |
| 401_contig_6196_SL-polyA | 605 | gi\|121998022\|ref\|YP_001002809.1\|acyl carrier protein [*Halorhodospira halophila* SL1] | 77 | 2 |
| 401_contig_15584 | 2683 | gi\|545373212\|ref\|XP_005650993.1\|ketoacyl-synt-domain-containing protein [*Coccomyxa subellipsoidea* C-169] | 69 | -1 |
| 401_contig_18967 | 429 | gi\|58613407\|gb\|AAW79290.1\|chloroplast acyl carrier protein [*Heterocapsa triquetra*] | 69 | 2 |
| 401_contig_21086 | 560 | gi\|58613407\|gb\|AAW79290.1\|chloroplast acyl carrier protein [*Heterocapsa triquetra*] | 58 | -1 |
| 401_contig_30745 | 643 | gi\|58613407\|gb\|AAW79290.1\|chloroplast acyl carrier protein [*Heterocapsa triquetra*] | 81 | 2 |
| 401_contig_39409 | 3132 | gi\|113476399\|ref\|YP_722460.1\|hypothetical protein Tery_2811 [*Trichodesmium erythraeum* IMS101] | 42 | 2 |
| 401_contig_41147 | 593 | gi\|58613407\|gb\|AAW79290.1\|chloroplast acyl carrier protein [*Heterocapsa triquetra*] | 78 | -1 |
| 401_contig_44538 | 1356 | gi\|255085504\|ref\|XP_002505183.1\|predicted protein [*Micromonas* sp. RCC299] | 63 | -2 |
| 401_contig_2244 | 3693 | gi\|156356414\|ref\|XP_001623919.1\|predicted protein [*Nematostella vectensis*] | 45 | 2 |
| 401_contig_13205 | 2549 | gi\|156342046\|ref\|XP_001620860.1\|hypothetical protein NEMVEDRAFT_v1g222634 [*Nematostella vectensis*] | 44 | 2 |
| 401_contig_22540 | 2881 | gi\|156356406\|ref\|XP_001623915.1\|predicted protein [*Nematostella vectensis*] | 42 | -2 |
| 401_contig_36888 | 2681 | gi\|156396813\|ref\|XP_001637587.1\|predicted protein [*Nematostella vectensis*] | 43 | -1 |
| 401_contig_40213 | 2956 | gi\|156356406\|ref\|XP_001623915.1\|predicted protein [*Nematostella vectensis*] | 44 | 3 |
| 401_contig_53119 | 2680 | gi\|156396813\|ref\|XP_001637587.1\|predicted protein [*Nematostella vectensis*] | 48 | 3 |
| 149_contig_10818_polyA | 586 | gi\|58613407\|gb\|AAW79290.1\|chloroplast acyl carrier protein [*Heterocapsa triquetra*] | 77 | 2 |
| 149_contig_13574 | 569 | gi\|58613407\|gb\|AAW79290.1\|chloroplast acyl carrier protein [*Heterocapsa triquetra*] | 79 | 3 |
| 149_contig_17402_polyA | 1139 | gi\|58613407\|gb\|AAW79290.1\|chloroplast acyl carrier protein [*Heterocapsa triquetra*] | 70 | 3 |
| 149_contig_19277 | 671 | gi\|58613407\|gb\|AAW79290.1\|chloroplast acyl carrier protein [*Heterocapsa triquetra*] | 60 | -3 |
| 149_contig_19874 | 2603 | gi\|156342046\|ref\|XP_001620860.1\|hypothetical protein NEMVEDRAFT_v1g222634 [*Nematostella vectensis*] | 41 | 3 |
| 149_contig_20449_polyA | 530 | gi\|58613407\|gb\|AAW79290.1\|chloroplast acyl carrier protein [*Heterocapsa triquetra*] | 77 | -2 |
| 149_contig_27511 | 683 | gi\|58613407\|gb\|AAW79290.1\|chloroplast acyl carrier protein [*Heterocapsa triquetra*] | 75 | 2 |
| 149_contig_84241_polyA | 422 | gi\|428174268\|gb\|EKX43165.1\|hypothetical protein GUITHDRAFT_110892 [*Guillardia theta* CCMP2712] | 61 | 1 |
| 149_contig_87156 | 604 | gi\|485610972\|gb\|EOD09665.1\|polyketide synthase [*Emiliania huxleyi* CCMP1516] | 68 | 1 |
| 149_contig_841 | 2892 | gi\|156356414\|ref\|XP_001623919.1\|predicted protein [*Nematostella vectensis*] | 47 | 2 |
| 149_contig_7448 | 2692 | gi\|156356406\|ref\|XP_001623915.1\|predicted protein [*Nematostella vectensis*] | 43 | 2 |
| 149_contig_13429 | 3373 | gi\|156342046\|ref\|XP_001620860.1\|hypothetical protein NEMVEDRAFT_v1g222634 [*Nematostella vectensis*] | 44 | 2 |
| 149_contig_43235 | 2689 | gi\|545373212\|ref\|XP_005650993.1\|ketoacyl-synt-domain-containing protein [*Coccomyxa subellipsoidea* C-169] | 55 | -3 |

Supplementary table 10: Sequence properties of the transcripts encoding epoxidases, epoxide hydrolases and full and partial sulfotransferases enzymes identified in *G. australes* (sequences name starting with 149) and *G. belizeanus* (sequences name starting with 401).

| **Seq. Name** | **Seq. Length** | **Blast Hit Description (HSP)** | **Similarity (%)** | **Query Frame** |
| --- | --- | --- | --- | --- |
| **Epoxidases** | | | | |
| 149_contig_23581 | 2039 | gi\|298714131\|emb\|CBJ27312.1\|zeaxanthin epoxidase, chloroplast precursor [*Ectocarpus siliculosus*] | 66 | -3 |
| 149_contig_16625 | 2372 | gi\|223995267\|ref\|XP_002287317.1\|zeaxanthin epoxidase [*Thalassiosira pseudonana* CCMP1335] | 78 | -2 |
| 149_contig_19107 | 1945 | gi\|224006239\|ref\|XP_002292080.1\|diadinoxanthin de-epoxidase [*Thalassiosira pseudonana* CCMP1335] | 63 | -1 |
| 401_contig_3482 | 2507 | gi\|223995267\|ref\|XP_002287317.1\|zeaxanthin epoxidase [*Thalassiosira pseudonana* CCMP1335] | 79 | -1 |
| 401_contig_4945 | 1923 | gi\|298714131\|emb\|CBJ27312.1\|zeaxanthin epoxidase, chloroplast precursor [*Ectocarpus siliculosus*] | 67 | 3 |
| 401_contig_28889 | 1609 | gi\|224006239\|ref\|XP_002292080.1\|diadinoxanthin de-epoxidase [*Thalassiosira pseudonana* CCMP1335] | 64 | -3 |
| 401_contig_67156 | 1542 | gi\|255073057\|ref\|XP_002500203.1\|violaxanthin de-epoxidase [*Micromonas* sp. RCC299] | 52 | 2 |
| **Epoxide hydrolases** | | | | |
| 149_contig_9520 | 1451 | gi\|384249393\|gb\|EIE22875.1\|epocide hydrolase domain-containing protein [*Coccomyxa subellipsoidea* C-169] | 60 | -3 |
| 149_contig_71956 | 1202 | gi\|260062305\|ref\|YP_003195385.1\|epoxide hydrolase [*Robiginitalea biformata* HTCC2501] | 50 | 3 |
| 149_contig_86217 | 853 | gi\|448545839\|ref\|ZP_21626250.1\|epoxide hydrolase-like protein yfhM [*Haloferax* sp. ATCC BAA-646] | 48 | -1 |
| 149_contig_39874 | 588 | gi\|147904364\|ref\|NP_001087143.1\|epoxide hydrolase 2, cytoplasmic [*Xenopus laevis*] | 68 | -1 |
| 149_contig_97081 | 424 | gi\|395531456\|ref\|XP_003767794.1\|PREDICTED: epoxide hydrolase 1 [*Sarcophilus harrisii*] | 61 | 3 |
| 149_contig_32778_polyA | 1200 | gi\|209155350\|gb\|ACI33907.1\|Epoxide hydrolase 2 [*Salmo salar*] | 57 | -3 |
| 401_contig_9239 | 1240 | gi\|323454747\|gb\|EGB10616.1\|hypothetical protein AURANDRAFT_21856 [*Aureococcus anophagefferens*] | 56 | -1 |
| 401_contig_8594 | 1382 | gi\|384249393\|gb\|EIE22875.1\|epocide hydrolase domain-containing protein [*Coccomyxa subellipsoidea* C-169] | 60 | 1 |
| 401_contig_42706 | 1268 | gi\|260062305\|ref\|YP_003195385.1\|epoxide hydrolase [*Robiginitalea biformata* HTCC2501] | 50 | 1 |
| 401_contig_60138 | 1224 | gi\|209155350\|gb\|ACI33907.1\|Epoxide hydrolase 2 [*Salmo salar*] | 55 | 3 |
| **Sulfotransferases** | | | | |
| 149_contig_376 | 1824 | gi\|299116384\|emb\|CBN74649.1\|Sulfotransferase [*Ectocarpus siliculosus*] | 44 | -1 |
| 149_contig_6948_polyA | 1231 | gi\|167518043\|ref\|XP_001743362.1\|hypothetical protein [*Monosiga brevicollis* MX1] | 47 | 2 |
| 149_contig_7964_sl | 919 | gi\|156383419\|ref\|XP_001632831.1\|predicted protein [*Nematostella vectensis*] | 47 | -2 |
| 149_contig_18361 | 1378 | gi\|494034010\|ref\|WP_006976143.1\|Sulfotransferase [*Plesiocystis pacifica*] | 44 | -1 |
| 149_contig_18524 | 1287 | gi\|323453637\|gb\|EGB09508.1\|hypothetical protein AURANDRAFT_63144 [*Aureococcus anophagefferens*] | 47 | 2 |
| 149_contig_18761 | 1405 | gi\|485641282\|gb\|EOD35448.1\|hypothetical protein EMIHUDRAFT_98474 [*Emiliania huxleyi* CCMP1516] | 48 | 1 |
| 149_contig_19699 | 1033 | gi\|159472246\|ref\|XP_001694262.1\|hypothetical protein CHLREDRAFT_173868 [*Chlamydomonas reinhardtii*] | 45 | -2 |
| 149_contig_22759_sl | 1386 | gi\|409993097\|ref\|ZP_11276252.1\|sulfotransferase [*Arthrospira platensis* str. Paraca] | 46 | 1 |
| 149_contig_27765 | 1443 | gi\|299116384\|emb\|CBN74649.1\|Sulfotransferase [*Ectocarpus siliculosus*] | 46 | -3 |
| 149_contig_41667 | 1056 | gi\|494034010\|ref\|WP_006976143.1\|Sulfotransferase [*Plesiocystis pacifica*] | 47 | 2 |
| 149_contig_42037 | 1154 | gi\|497836687\|ref\|WP_010150843.1\|alcohol sulfotransferase [SAR324 cluster bacterium JCVI-SC AAA005] | 49 | -2 |
| 149_contig_44410 | 1111 | gi\|262195957\|ref\|YP_003267166.1\|sulfotransferase [*Haliangium ochraceum* DSM 14365] | 49 | 1 |
| 149_contig_54621 | 1275 | gi\|262193808\|ref\|YP_003265017.1\|sulfotransferase [*Haliangium ochraceum* DSM 14365] | 45 | 2 |
| 149_contig_59139 | 1088 | gi\|209525653\|ref\|ZP_03274190.1\|sulfotransferase [*Arthrospira maxima* CS-328] | 46 | 3 |
| 149_contig_64819 | 509 | gi\|262193808\|ref\|YP_003265017.1\|sulfotransferase [*Haliangium ochraceum* DSM 14365] | 71 | 3 |
| 149_contig_72035 | 1245 | gi\|323450808\|gb\|EGB06687.1\|hypothetical protein AURANDRAFT_65313 [*Aureococcus anophagefferens*] | 47 | -1 |
| 149_contig_88811 | 763 | gi\|494034010\|ref\|WP_006976143.1\|Sulfotransferase [*Plesiocystis pacifica*] | 41 | -2 |
| 401_contig_1108 | 1147 | gi\|340374041\|ref\|XP_003385547.1\|PREDICTED: sulfotransferase 1C2A-like [*Amphimedon queenslandica*] | 54 | 1 |
| 401_contig_1139 | 1277 | gi\|428215444\|ref\|YP_007088588.1\|sulfotransferase family protein [*Oscillatoria acuminata* PCC 6304] | 45 | 1 |
| 401_contig_1349 | 1155 | gi\|159472246\|ref\|XP_001694262.1\|hypothetical protein CHLREDRAFT_173868 [*Chlamydomonas reinhardtii*] | 45 | -1 |
| 401_contig_11332_polyA | 1270 | gi\|442323606\|ref\|YP_007363627.1\|hypothetical protein MYSTI_06670 [*Myxococcus stipitatus* DSM 14675] | 50 | -3 |
| 401_contig_16824 | 3247 | gi\|310821364\|ref\|YP_003953722.1\|hypothetical protein STAUR_4111 [*Stigmatella aurantiaca* DW4/3-1] | 51 | 1 |
| 401_contig_20359 | 949 | gi\|340378497\|ref\|XP_003387764.1\|PREDICTED: amine sulfotransferase-like [*Amphimedon queenslandica*] | 51 | 3 |
| 401_contig_22004 | 1428 | gi\|299116384\|emb\|CBN74649.1\|Sulfotransferase [*Ectocarpus siliculosus*] | 47 | -1 |
| 401_contig_24113 | 454 | gi\|514655520\|ref\|XP_004999048.1\|hypothetical protein PTSG_11596 [*Salpingoeca* sp. ATCC 50818] | 60 | 3 |
| 401_contig_50067 | 505 | gi\|442323606\|ref\|YP_007363627.1\|hypothetical protein MYSTI_06670 [*Myxococcus stipitatus* DSM 14675] | 58 | 3 |
| 401_contig_53295 | 732 | gi\|241015529\|ref\|XP_002405640.1\|sulfotransferase, putative [*Ixodes scapularis*] | 55 | 2 |
| 401_contig_61880 | 629 | gi\|485641282\|gb\|EOD35448.1\|hypothetical protein EMIHUDRAFT_98474 [*Emiliania huxleyi* CCMP1516] | 51 | -2 |
| 401_contig_66092 | 727 | gi\|485626517\|gb\|EOD22232.1\|hypothetical protein EMIHUDRAFT_463686 [*Emiliania huxleyi* CCMP1516] | 53 | -2 |
| **Partial sulfotransferases** | | | | |
| 149_contig_18087 | 955 | gi\|402225153\|gb\|EJU05214.1\|hypothetical protein DACRYDRAFT_103708 [*Dacryopinax* sp. DJM-731 SS1] | 51 | -2 |
| 149_contig_19242 | 945 | gi\|485619052\|gb\|EOD15930.1\|hypothetical protein EMIHUDRAFT_210863 [*Emiliania huxleyi* CCMP1516] | 41 | 1 |
| 149_contig_21174 | 1381 | gi\|156395292\|ref\|XP_001637045.1\|predicted protein [*Nematostella vectensis*] | 45 | 2 |
| 149_contig_22175 | 1233 | gi\|291229596\|ref\|XP_002734759.1\|PREDICTED: carbohydrate sulfotransferase 14-like [*Saccoglossus kowalevskii*] | 43 | -2 |
| 149_contig_24622 | 1251 | gi\|485612596\|gb\|EOD10839.1\|hypothetical protein EMIHUDRAFT_215075 [*Emiliania huxleyi* CCMP1516] | 47 | -2 |
| 149_contig_26938 | 931 | gi\|405974261\|gb\|EKC38920.1\|Carbohydrate sulfotransferase 4 [*Crassostrea gigas*] | 45 | 2 |
| 149_contig_30732 | 1339 | gi\|156378568\|ref\|XP_001631214.1\|predicted protein [*Nematostella vectensis*] | 43 | 2 |
| 149_contig_31900 | 1203 | gi\|219114789\|ref\|XP_002178190.1\|predicted protein [*Phaeodactylum tricornutum* CCAP 1055/1] | 49 | 1 |
| 149_contig_36979 | 460 | gi\|323446025\|gb\|EGB02362.1\|hypothetical protein AURANDRAFT_68952 [*Aureococcus anophagefferens*] | 46 | -3 |
| 149_contig_50510 | 1671 | gi\|514694167\|ref\|XP_004994655.1\|hypothetical protein PTSG_04561 [*Salpingoeca* sp. ATCC 50818] | 45 | -2 |
| 149_contig_69981 | 1146 | gi\|504162960\|ref\|XP_004592043.1\|PREDICTED: galactosylceramide sulfotransferase [*Ochotona princeps*] | 50 | 1 |
| 149_contig_74974 | 1019 | gi\|323454385\|gb\|EGB10255.1\|hypothetical protein AURANDRAFT_62889 [*Aureococcus anophagefferens*] | 46 | -3 |
| 401_contig_2194_polyA | 1236 | gi\|296206043\|ref\|XP_002750040.1\|PREDICTED: galactose-3-O-sulfotransferase 2 [*Callithrix jacchus*] | 47 | 2 |
| 401_contig_3923 | 1068 | gi\|403291510\|ref\|XP_003936830.1\|PREDICTED: galactose-3-O-sulfotransferase 2 [*Saimiri boliviensis*] | 47 | 2 |
| 401_contig_5754 | 1167 | gi\|219110285\|ref\|XP_002176894.1\|predicted protein [*Phaeodactylum tricornutum* CCAP 1055/1] | 44 | -3 |
| 401_contig_8278 | 1001 | gi\|156408263\|ref\|XP_001641776.1\|predicted protein [*Nematostella vectensis*] | 43 | -2 |
| 401_contig_14161 | 1309 | gi\|483513634\|gb\|EOB01640.1\|Carbohydrate sulfotransferase 14, partial [*Anas platyrhynchos]* | 46 | -2 |
| 401_contig_21757 | 1104 | gi\|355747475\|gb\|EHH51972.1\|Carbohydrate sulfotransferase 12 [*Macaca fascicularis*] | 40 | -3 |
| 401_contig_46920 | 740 | gi\|321462001\|gb\|EFX73028.1\|hypothetical protein DAPPUDRAFT_110202 [*Daphnia pulex*] | 50 | -3 |
| 401_contig_46991 | 2908 | gi\|260942621\|ref\|XP_002615609.1\|hypothetical protein CLUG_04491 [*Clavispora lusitaniae* ATCC 42720] | 42 | 1 |
| 401_contig_50942_polyA | 514 | gi\|504165513\|ref\|XP_004592723.1\|PREDICTED: sulfotransferase 6B1-like [*Ochotona princeps*] | 50 | -1 |
| 401_contig_75289 | 1660 | gi\|514694167\|ref\|XP_004994655.1\|hypothetical protein PTSG_04561 [*Salpingoeca* sp. ATCC 50818] | 46 | 2 |
| 401_contig_110601 | 486 | gi\|397616992\|gb\|EJK64233.1\|hypothetical protein THAOC_15053 [*Thalassiosira oceanica*] | 50 | -3 |

Supplementary table 11: Sequence properties of the transcripts encoding full acyl carrier protein synthase, Enoyl reductase, acyl transferase, acyl carrier protein identified in *G. australes* (sequences name starting with 149) and *G. belizeanus* (sequences name starting with 401). These transcripts were considered as bacterial contaminants.

| **Seq. Name** | **Seq. Length** | **Blast Hit Description (HSP)** | **Similarity (%)** | **Query Frame** |
| --- | --- | --- | --- | --- |
| **Ketosynthase** | | | | |
| 149_contig_64398 | 1110 | gi\|55977013\|gb\|AAV68343.1\|type I polyketide synthase-like protein [unidentified microorganism] | 84 | 1 |
| 401_contig_14412 | 1442 | gi\|383455395\|ref\|YP_005369384.1\|malonyl CoA-acyl carrier protein transacylase [*Corallococcus coralloides*  DSM 2259] | 56 | 2 |
| 401_contig_77738 | 635 | gi\|494755674\|ref\|WP_007491082.1\|polyketide synthase [*Streptomyces zinciresistens*] | 49 | -3 |
| 401_contig_105365 | 719 | gi\|494433334\|ref\|WP_007226613.1\|beta-ketoacyl synthase [marine gamma proteobacterium HTCC2143] | 45 | 3 |
| 401_contig_109831 | 448 | gi\|489999772\|ref\|WP_003902753.1\|polyketide synthase pks2, partial [*Mycobacterium tuberculosis*] | 50 | -2 |
| 401_contig_38600 | 2686 | gi\|186682446\|ref\|YP_001865642.1\|beta-ketoacyl synthase [*Nostoc punctiforme* PCC 73102] | 49 | 1 |
| **Ketoreductases** | | | | |
| 401_contig_84442 | 1482 | gi\|186682448\|ref\|YP_001865644.1\|beta-ketoacyl synthase [*Nostoc punctiforme* PCC 73102] | 53 | 2 |
| 401_contig_108776 | 515 | gi\|108762545\|ref\|YP_632695.1\|polyketide synthase [*Myxococcus xanthus* DK 1622] | 60 | 3 |
| 401_contig_2043 | 6130 | gi\|515898619\|ref\|WP_017329202.1\|hypothetical protein [*Burkholderia pyrrocinia*] | 44 | 3 |
| 401_contig_107096 | 454 | gi\|186682448\|ref\|YP_001865644.1\|beta-ketoacyl synthase [*Nostoc punctiforme* PCC 73102] | 60 | -1 |
| 401_contig_26693 | 3795 | gi\|186682448\|ref\|YP_001865644.1\|beta-ketoacyl synthase [*Nostoc punctiforme* PCC 73102] | 52 | 2 |
| 401_contig_2297 | 1498 | gi\|499305043\|ref\|WP_010995818.1\| polyketide synthase [*Nostoc* sp. PCC 7120] | 56 | -3 |
| 401_contig_16029_polyA | 945 | gi\|442322941\|ref\|YP_007362962.1\|polyketide synthase [*Myxococcus stipitatus* DSM 14675] | 65 | 3 |
| 401_contig_59594_polyA | 563 | gi\|493394790\|ref\|WP_006350916.1\|beta-ketoacyl synthase [*Streptomyces tsukubaensis*] | 56 | -1 |
| 401_contig_54507 | 513 | gi\|517210958\|ref\|WP_018399776.1\|hypothetical protein [filamentous cyanobacterium ESFC-1] | 67 | -2 |
| 401_contig_78214 | 1107 | gi\|113477277\|ref\|YP_723338.1\|beta-ketoacyl synthase [*Trichodesmium erythraeum* IMS101] | 63 | -1 |
| 401_contig_58844 | 768 | gi\|498041874\|ref\|WP_010356030.1\|polyketide synthase [*Streptomyces acidiscabies*] | 47 | 3 |
| 149_contig_4536_polyA | 6047 | gi\|515898619\|ref\|WP_017329202.1\|hypothetical protein [*Burkholderia pyrrocinia*] | 46 | 1 |
| 149_contig_6983 | 5962 | gi\|488700098\|ref\|WP_002624106.1\|Malonyl CoA-acyl carrier protein transacylase [*Cystobacter fuscus*] | 51 | 3 |
| 149_contig_11987 | 1043 | gi\|442322941\|ref\|YP_007362962.1\|polyketide synthase [*Myxococcus stipitatus* DSM 14675] | 54 | 1 |
| 149_contig_17876 | 3193 | gi\|548436563\|ref\|WP_022524096.1\|Beta-ketoacyl synthase-like protein [*Halomonas* sp. A3H3] | 49 | 3 |
| 149_contig_17952_polyA | 831 | gi\|517210958\|ref\|WP_018399776.1\|hypothetical protein [filamentous cyanobacterium ESFC-1] | 67 | -3 |
| 149_contig_30859 | 5833 | gi\|497900914\|ref\|WP_010215070.1\|coronafacic acid polyketide synthase I, partial [*Pseudomonas syringae* group genomosp. 3] | 57 | 3 |
| 149_contig_38922 | 813 | gi\|436670011\|ref\|YP_007317750.1\|polyketide synthase family protein [*Cylindrospermum stagnale* PCC 7417] | 58 | 1 |
| 149_contig_48986_polyA | 5077 | gi\|161213731\|gb\|ABX60162.1\|polyketide synthase [*Cylindrospermopsis raciborskii* AWT205] | 54 | 2 |
| **Partial ketoreductases** | | | | |
| 149_contig_7193_polyA | 2266 | gi\|182439974\|ref\|YP_001827693.1\|type-I PKS [*Streptomyces griseus* subsp. griseus NBRC 13350] | 64 | 2 |
| 149_contig_42343 | 736 | gi\|37521523\|ref\|NP_924900.1\|polyketide synthase [*Gloeobacter violaceus* PCC 7421] | 48 | 1 |
| 149_contig_69710 | 1271 | gi\|94467039\|dbj\|BAE93729.1\|type I polyketide synthase [*Streptomyces* sp. NRRL 11266] | 44 | -1 |
| 149_contig_75540 | 423 | gi\|75914738\|gb\|ABA29781.1\|MmxC [*Cystobacter fuscus*] | 58 | -2 |
| 149_contig_92721 | 433 | gi\|521464319\|ref\|YP_008151403.1\|hypothetical protein SCE1572_25010 [*Sorangium cellulosum* So0157-2] | 56 | 2 |
| 149_contig_98798 | 406 | gi\|545903977\|ref\|WP_021780892.1\|Malonyl CoA-acyl carrier protein transacylase protein [*Myxococcus* sp. (contaminant ex DSM 436)] | 69 | -3 |
| **Acyltransferases** | | | | |
| 149_contig_18693 | 1260 | gi\|498272758\|ref\|WP_010586914.1\|malonyl CoA-ACP transacylase [*Schlesneria paludicola*] | 51 | -3 |
| 149_contig_40178 | 1268 | gi\|490510491\|ref\|WP_004376497.1\|ACP S-malonyltransferase [*Prevotella oris*] | 50 | 2 |
| **Acyltransferases protein synthases** | | | | |
| 401_congig_47080_polyA | 1743 | gi\|515856584\|ref\|WP_017287212.1\|3-oxoacyl-ACP synthase [*Leptolyngbya boryana*] | 61 | -2 |
| **Acyl carrier proteins** | | | | |
| 401_contig_10664 | 2750 | gi\|493388409\|ref\|WP_006344600.1\|type I modular polyketide synthase, partial [*Streptomyces tsukubaensis*] | 57 | 1 |
| 401_contig_56405 | 1312 | gi\|428203301\|ref\|YP_007081890.1\|polyketide synthase family protein [*Pleurocapsa* sp. PCC 7327] | 70 | 2 |
| 401_contig_77950 | 782 | gi\|494035178\|ref\|WP_006977307.1\|putative type I polyketide synthase, partial [*Plesiocystis pacifica*] | 71 | -3 |
| 401_contig_87229 | 624 | gi\|216409660\|dbj\|BAH02269.1\|polyketide synthase [*Streptomyces platensis*] | 57 | -2 |
| 401_contig_468 | 763 | gi\|491160492\|ref\|WP_005018879.1\|acyl carrier protein [*Acinetobacter radioresistens*] | 57 | -3 |
| 401_contig_1565 | 2770 | gi\|113476399\|ref\|YP_722460.1\|hypothetical protein Tery_2811 [*Trichodesmium erythraeum* IMS101] | 43 | 3 |
| 401_contig_2244 | 3693 | gi\|307150987\|ref\|YP_003886371.1\|hypothetical protein Cyan7822_1089 [*Cyanothece* sp. PCC 7822] | 44 | 2 |
| 401_contig_5388 | 2631 | gi\|218437859\|ref\|YP_002376188.1\|hypothetical protein PCC7424_0864 [*Cyanothece* sp. PCC 7424] | 43 | -1 |
| 401_contig_7495 | 2264 | gi\|408676205\|ref\|YP_006876032.1\|Malonyl CoA-acyl carrier protein transacylase [*Streptomyces venezuelae* ATCC 10712] | 48 | -2 |
| 401_contig_13205 | 2549 | gi\|495332491\|ref\|WP_008057229.1\|hypothetical protein [*Arthrospira* sp. PCC 8005] | 42 | 2 |
| 401_contig_22540 | 2881 | gi\|493031743\|ref\|WP_006101140.1\|Tetratricopeptide repeat family [*Coleofasciculus chthonoplastes*] | 51 | -2 |
| 401_contig_36888 | 2681 | gi\|479127724\|ref\|YP_005067684.1\|TPR domain protein [*Arthrospira platensis* NIES-39] | 45 | -1 |
| 401_contig_38856 | 2643 | gi\|113476399\|ref\|YP_722460.1\|hypothetical protein Tery_2811 [*Trichodesmium erythraeum* IMS101] | 45 | 1 |
| 401_contig_40213 | 2956 | gi\|156356406\|ref\|XP_001623915.1\|predicted protein [*Nematostella vectensis*] | 44 | 3 |
| 401_contig_40213 | 2956 | gi\|113476399\|ref\|YP_722460.1\|hypothetical protein Tery_2811 [*Trichodesmium erythraeum* IMS101] | 47 | 3 |
| 401_contig_45792 | 940 | gi\|407648744\|ref\|YP_006812503.1\|non-ribosomal peptide synthetase [Nocardia brasiliensis ATCC 700358] | 49 | 2 |
| 401_contig_53119 | 2680 | gi\|493719492\|ref\|WP_006668997.1\|hypothetical protein [*Arthrospira maxima*] | 43 | 3 |
| 401_contig_63472_polyA | 1630 | gi\|383455396\|ref\|YP_005369385.1\|malonyl CoA-acyl carrier protein transacylase [*Corallococcus coralloides*  DSM 2259] | 59 | -2 |
| 149_contig_1419 | 2750 | gi\|113476399\|ref\|YP_722460.1\|hypothetical protein Tery_2811 [*Trichodesmium erythraeum* IMS101] | 44 | -3 |
| 149_contig_19874 | 2603 | gi\|156342046\|ref\|XP_001620860.1\|hypothetical protein NEMVEDRAFT_v1g222634 [*Nematostella vectensis*] | 41 | 3 |
| 149_contig_2460 | 2699 | gi\|428225222\|ref\|YP_007109319.1\|hypothetical protein GEI7407_1780 [*Geitlerinema* sp. PCC 7407] | 46 | 3 |
| 149_contig_8376 | 2936 | gi\|479127724\|ref\|YP_005067684.1\|TPR domain protein [*Arthrospira platensis* NIES-39] | 43 | 3 |
| 149_contig_11830 | 2593 | gi\|307152085\|ref\|YP_003887469.1\|hypothetical protein Cyan7822_2215 [*Cyanothece* sp. PCC 7822] | 43 | 3 |
| 149_contig_29369 | 2717 | gi\|113476399\|ref\|YP_722460.1\|hypothetical protein Tery_2811 [*Trichodesmium erythraeum* IMS101] | 44 | -1 |
| 149_contig_45849 | 2807 | gi\|113476399\|ref\|YP_722460.1\|hypothetical protein Tery_2811 [*Trichodesmium erythraeum* IMS101] | 47 | 3 |
| **Thioesterases** | | | | |
| 149_contig_27369\|oleoyl-(acyl-carrier-protein) | 1450 | gi\|493076138\|ref\|WP_006123301.1\|thioesterase [*Streptomyces filamentosus*] | 37 | 2 |

Supplementary table 12: Sequence properties of the transcripts encoding epoxide hydrolases and sulfotransferases enzymes identified in G. australes (sequence name starting with 149) and *G. belizeanus* (sequences name starting with 401). These sequences were considered as bacterial contaminants.

| **Sequence Name** | **Seq. Length** | **Blast Hit Description (HSP)** | **Similarity (%)** | **Query Frame** |
| --- | --- | --- | --- | --- |
| **Sulfotransferases** | | | | |
| 149_contig_18087 | 955 | gi\|402225153\|gb\|EJU05214.1\|hypothetical protein DACRYDRAFT_103708 [*Dacryopinax* sp. DJM-731 SS1] | 51 | -2 |
| 149_contig_19242 | 945 | gi\|485619052\|gb\|EOD15930.1\|hypothetical protein EMIHUDRAFT_210863 [*Emiliania huxleyi* CCMP1516] | 41 | 1 |
| 149_contig_21174 | 1381 | gi\|156395292\|ref\|XP_001637045.1\|predicted protein [*Nematostella vectensis*] | 45 | 2 |
| 149_contig_22175 | 1233 | gi\|291229596\|ref\|XP_002734759.1\|PREDICTED: carbohydrate sulfotransferase 14-like [*Saccoglossus kowalevskii*] | 43 | -2 |
| 149_contig_24622 | 1251 | gi\|485612596\|gb\|EOD10839.1\|hypothetical protein EMIHUDRAFT_215075 [*Emiliania huxleyi* CCMP1516] | 47 | -2 |
| 149_contig_26938 | 931 | gi\|405974261\|gb\|EKC38920.1\|Carbohydrate sulfotransferase 4 [*Crassostrea gigas*] | 45 | 2 |
| 149_contig_30732 | 1339 | gi\|156378568\|ref\|XP_001631214.1\|predicted protein [*Nematostella vectensis*] | 43 | 2 |
| 149_contig_31900 | 1203 | gi\|219114789\|ref\|XP_002178190.1\|predicted protein [*Phaeodactylum tricornutum* CCAP 1055/1] | 49 | 1 |
| 149_contig_36979 | 460 | gi\|323446025\|gb\|EGB02362.1\|hypothetical protein AURANDRAFT_68952 [*Aureococcus anophagefferens*] | 46 | -3 |
| 149_contig_50510 | 1671 | gi\|514694167\|ref\|XP_004994655.1\|hypothetical protein PTSG_04561 [*Salpingoeca* sp. ATCC 50818] | 45 | -2 |
| 149_contig_69981 | 1146 | gi\|504162960\|ref\|XP_004592043.1\|PREDICTED: galactosylceramide sulfotransferase [*Ochotona princeps*] | 50 | 1 |
| 149_contig_74974 | 1019 | gi\|323454385\|gb\|EGB10255.1\|hypothetical protein AURANDRAFT_62889 [*Aureococcus anophagefferens*] | 46 | -3 |
| 401_contig_603 | 1363 | gi\|494034010\|ref\|WP_006976143.1\|Sulfotransferase [*Plesiocystis pacifica*] | 42 | 3 |
| 401_contig_6612 | 1259 | gi\|156378568\|ref\|XP_001631214.1\|predicted protein [*Nematostella vectensis*] | 45 | 1 |
| 401_contig_15749 | 1103 | gi\|497836687\|ref\|WP_010150843.1\|alcohol sulfotransferase [SAR324 cluster bacterium JCVI-SC AAA005] | 67 | -3 |
| 401_contig_26354 | 1339 | gi\|159472246\|ref\|XP_001694262.1\|hypothetical protein CHLREDRAFT_173868 [*Chlamydomonas reinhardtii*] | 46 | -3 |
| 401_contig_32338 | 686 | gi\|516324822\|ref\|WP_017715481.1\|hypothetical protein [*Oscillatoria* sp. PCC 10802] | 54 | -1 |
| 401_contig_34864 | 1064 | gi\|485612596\|gb\|EOD10839.1\|hypothetical protein EMIHUDRAFT_215075 [*Emiliania huxleyi* CCMP1516] | 46 | -1 |
| 401_contig_54307 | 688 | gi\|218441112\|ref\|YP_002379441.1\|sulfotransferase [*Cyanothece* sp*.* PCC 7424] | 48 | 3 |
| 401_contig_60204 | 1056 | gi\|497994744\|ref\|WP_010308900.1\|sulfotransferase [*Synechococcus* sp. CB0101] | 53 | 2 |
| 401_contig_61112 | 569 | gi\|435854908\|ref\|YP_007316227.1\|sulfotransferase family protein [*Halobacteroides halobius* DSM 5150] | 54 | -2 |
| 401_contig_62273 | 713 | gi\|428215444\|ref\|YP_007088588.1\|sulfotransferase family protein [*Oscillatoria acuminata* PCC 6304] | 53 | 1 |
| 401_contig_64927 | 1265 | gi\|323450808\|gb\|EGB06687.1\|hypothetical protein AURANDRAFT_65313 [*Aureococcus anophagefferens*] | 48 | -2 |
| 401_contig_66444 | 1853 | gi\|522030845\|ref\|WP_020542054.1\|hypothetical protein [*Nonomuraea coxensis*] | 43 | -2 |
| 401_contig_82120 | 468 | gi\|435854908\|ref\|YP_007316227.1\|sulfotransferase family protein [*Halobacteroides halobius* DSM 5150] | 55 | 3 |
| 401_contig_89834 | 778 | gi\|497836687\|ref\|WP_010150843.1\|alcohol sulfotransferase [SAR324 cluster bacterium JCVI-SC AAA005] | 53 | 1 |
| **Epoxide hydrolases** | | | | |
| 401_contig_14032 | 1480 | gi\|404422215\|ref\|ZP_11003911.1\|epoxide hydrolase [*Mycobacterium fortuitum* subsp. fortuitum DSM 46621] | 61 | 3 |

Supplementary table 13: Sequence properties of the transcripts encoding multiple Type I PKS domains identified in *G. australes* (sequence name starting with 149) and *G. belizeanus* (sequence name starting with 401). These sequences were considered as bacterial contaminants. KS-ketosynthase domain; KR-ketoreductase domain; AT- acyltransferase domain; ACPS- acyl carrier protein synthase; ACP- acyl carrier protein domain; TE- thioesterase domain; MT- methyl transferase domain; DH- dehydratase domain; ER- enoylreductase domain.

| **Seq. Name** | **Seq. Length** | **Blast Hit Description (HSP)** | **Similarity (%)** | **Query Frame** |
| --- | --- | --- | --- | --- |
| **KR-MT** | | | | |
| 149_contig_4536_polyA | 6047 | gi\|442322951\|ref\|YP_007362972.1\|polyketide synthase [*Myxococcus stipitatus* DSM 14675] | 58 | 1 |
| 149_contig_6983 | 5962 | gi\|488700098\|ref\|WP_002624106.1\|Malonyl CoA-acyl carrier protein transacylase [*Cystobacter fuscus*] | 51 | -3 |
| 149_contig_30859 | 5833 | gi\|497900914\|ref\|WP_010215070.1\|coronafacic acid polyketide synthase I, partial [*Pseudomonas syringae* group genomosp. 3] | 57 | -3 |
| 149_contig_48986_polyA | 5077 | gi\|161213731\|gb\|ABX60162.1\|polyketide synthase [*Cylindrospermopsis raciborskii* AWT205] | 54 | 2 |
| 401_contig_26693 | 3795 | gi\|186682448\|ref\|YP_001865644.1\|beta-ketoacyl synthase [*Nostoc punctiforme* PCC 73102] | 52 | 2 |
| **AT-TE-KR-ACP** | | | | |
| 401_contig_45222 | 4771 | gi\|491084742\|ref\|WP_004946356.1\|thiotemplate mechanism natural product synthetase [*Streptomyces mobaraensis*] | 49 | 2 |
| **ACP-KS-AT-DH-ER-KR-ACP-TE** | | | | |
| 149_contig_20703 | 7177 | gi\|95007112\|emb\|CAJ20333.1\|type I fatty acid synthase, putative [*Toxoplasma gondii* RH] | 57 | 2 |
|  |  | BLASTX for only KS domain sequence- malonyl CoA-acyl carrier protein transacylase [*Corallococcus coralloides*  DSM 2259] | 49 | 2 |
| **DH-ER-KR-ACP-TE** | | | | |
| 149_contig_45392 | 5340 | gi\|401395940\|ref\|XP_003879716.1\|short-chain dehydrogenase/reductase SDR, related [*Neospora caninum* Liverpool] | 50 | 1 |
|  |  | BLASTX for only KR domain sequence- >gb\|EFL26022.1\|modular polyketide synthase [*Streptomyces himastatinicus* ATCC 53653] | 48 | 1 |
| 401_contig_11718 | 5224 | gi\|401395940\|ref\|XP_003879716.1\|short-chain dehydrogenase/reductase SDR, related [*Neospora caninum* Liverpool] | 50 | -3 |
|  |  | BLASTX for only KR domain sequence- >gb\|EFL26022.1\|modular polyketide synthase [*Streptomyces himastatinicus* ATCC 53653] | 40 | -3 |
| **KR-DH-ER-ACP** | | | | |
| 401_contig_32761 | 4250 | gi\|169977289\|emb\|CAQ18830.1\|polyketide synthase [*Chondromyces crocatus*] | 58 | -2 |
|  |  | BLASTX for only KR domain sequence- beta-ketoacyl synthase [*Nostoc punctiforme* PCC 73102] | 37 | -2 |
| **KR-ACP-TE** | | | | |
| 401_contig_79005 | 2027 | gi\|401395940\|ref\|XP_003879716.1\|short-chain dehydrogenase/reductase SDR, related [*Neospora caninum* Liverpool] | 55 | 3 |
|  |  | BLASTX for only KR domain sequence- >gb\|EFL26022.1\|modular polyketide synthase [*Streptomyces himastatinicus* ATCC 53653] | 43 | 3 |

**Supplemental References**

1. Veldhuis, M.J.W., Cucci, T.L., and Sieracki, M.E. (1997). Cellular DNA content of marine phytoplankton using two new fluorochromes: taxonomic and ecological Implications. J. Phycol. *33*, 527-541.

2. LaJeunesse, T.C., Lambert, G., Andersen, R.A., Coffroth, M.A., and Galbraith, D.W. (2005). *Symbiodinium* (Pyrrhophyta) genome sizes (DNA content) are smallest among dinoflagellates. J. Phycol. *41*, 880-886.

3. Shoguchi, E., Shinzato, C., Kawashima, T., Gyoja, F., Mungpakdee, S., Koyanagi, R., Takeuchi, T., Hisata, K., Tanaka, M., Fujiwara, M., et al. (2013). Draft Assembly of the *Symbiodinium minutum* Nuclear Genome Reveals Dinoflagellate Gene Structure. Curr. Biol. *23*, 1399-1408.
